# Germline mutations predisposing to diffuse large B-cell lymphoma

**DOI:** 10.1038/bcj.2017.15

**Published:** 2017-02-17

**Authors:** O C Leeksma, N F de Miranda, H Veelken

**Affiliations:** 1 Department of Hematology/Medical Oncology, Onze Lieve Vrouwe Gasthuis, Amsterdam, The Netherlands; 2 Department of Hematology, Leiden University Medical Center, Leiden, The Netherlands; 3 Department of Pathology, Leiden University Medical Center, Leiden, The Netherlands

## Abstract

Genetic studies of diffuse large B-cell lymphomas (DLBCLs) in humans have revealed numerous targets of somatic mutations and an increasing number of potentially relevant germline alterations. The latter often affect genes involved in DNA repair and/or immune function. In general, defects in these genes also predispose to other conditions. Knowledge of these mutations can lead to disease-preventing measures in the patient and relatives thereof. Conceivably, these germline mutations will be taken into account in future therapy of the lymphoma. In other hematological malignancies, mutations originally found as somatic aberrations have also been shown to confer predisposition to these diseases, when occurring in the germline. Further interrogations of the genome in DLBCL patients are therefore expected to reveal additional hereditary predisposition genes. Our review shows that germline mutations have already been described in over one-third of the genes that are somatically mutated in DLBCL. Whether such germline mutations predispose carriers to DLBCL is an open question. Symptoms of the inherited syndromes associated with these genes range from anatomical malformations to intellectual disability, immunodeficiencies and malignancies other than DLBCL. Inherited or *de novo* alterations in protein-coding and non-coding genes are envisioned to underlie this lymphoma.

## Introduction

With an age-adjusted incidence of 7 per 100 000 individuals in the United States^1^ and 3.8 per 100 000 individuals in Europe,^2^ diffuse large B-cell lymphoma (DLBCL) has the highest incidence of any hematological malignancy. Although the prognosis of patients diagnosed with DLBCL has improved considerably by the introduction of immunochemotherapy, still 30–40% of the affected patients will eventually die from this disease.

As holds true for cancer in general, a combination of inherited and environmental factors can lead to the development of DLBCL. These B-cell lymphomas differ from many other cancers because of the role of their cells of origin in the immune system. The malignant transformation of these cells largely relies on the same genetic mechanisms that physiologically optimize their immunoglobulin antigen receptor (V(D)J gene recombination, class switch recombination and somatic hypermutation).^3^ These very dynamic processes require a high-fidelity DNA repair system. No wonder inherited defects in genes involved in DNA damage responses were shown to predispose to this disease (often via incapacitation of appropriate anti-viral, in particular epstein barr virus (EBV), responses by simultaneously affecting T cells, which use an identical enzymatic machinery to generate antigen specific T-cell receptors). Presumably together with an underlying defect in the non-homologous end joining (NHEJ) repair pathway, enzymatic activity of RAG1, RAG2 and activation-induced cytidine deaminase (AID) needed for antibody diversification and B-cell antigen receptor refinement can erroneously juxtapose oncogenes (such as *BCL2* or *MYC*) or immune checkpoint genes (such as *PD-L1*) to immunoglobulin genes.^3^ These immunoglobin gene translocation partners then use the immunoglobulin heavy-chain gene promotor for their overexpression to induce survival and growth or immune escape. AID can, in addition, induce off-target mutations by aberrant somatic hypermutation.^4, 5, 6, 7, 8^ The enzyme deaminates cytosine into uracil in single-stranded DNA. Uracil:Guanine mismatches either result in double-strand breaks or mutations. How AID is targeted to DNA outside of the Ig-loci is not completely resolved. The enzyme may be misguided to super-enhancer sequences^9^ and non-coding RNAs appear to be essential for the recognition of target DNA motifs.^10^ Chronic antigenic stimulation can also promote genomic instability in rapidly dividing B cells and AID-dependent lymphoma.^11^ Antibodies, but perhaps also other antigen receptors, may diversify under these circumstances beyond the classical V(D)J recombination via interchromosomal DNA insertions encoding antigen-recognizing protein sequences.^12^ Finally, infections with the human immunodeficiency virus can cause an immunosurveillance failure facilitating other infections and DLBCL.

Next-generation sequencing of DNA and RNA has been primarily used to identify potentially targetable somatic mutations in DLBCL to improve treatment. These studies have yielded a wealth of information on the genetic landscape of DLBCL. Most studies compared lymphoma sequences with peripheral blood sequences to discard germline variants.^5, 7, 8, 13^ On the basis of number of somatic mutations detected, DLBCL is a very heterogeneous disease, although the functional consequence of many of these mutations still needs to be resolved. In the majority of these studies, whole-exome sequencing was performed and a limited number of germline variations were actually reported. A whole-genome study by Morin *et al.* revealed that DLBCL is not necessarily the consequence of a gradual accumulation of chromosomal translocations and somatic mutations.^8^ It may also result as shown previously in Chronic Lymphocytic Leukemia and other cancers from chromothripsis, a single genomic catastrophe in which chromosomes are shattered into pieces and chaotically repaired.^14^

Knowledge on inherited mutations predisposing to DLBCL largely comes from studies of patients with immunodeficiencies. A better understanding of the mechanisms underlying these immune disorders has led to the identification of several genes, in which germline mutations promote the development of DLBCL and other cancers.

As has been observed in other malignancies, an increase in genetic screenings will reveal that mutations originally found somatically in DLBCL may also occur in the germline of patients. We will first review the literature, including supplementary files not readily retrievable by PubMed searches, on inherited or *de novo* pathogenic germline alterations in DLBCL patients. Second, our analysis of somatically mutated genes will illustrate that it is conceivable that many of the somatic mutations in DLBCL also predispose to this disease when occurring in the germ line.

## Germline mutations in DLBCL

Forty-nine genes with a (presumed) causative role in lymphomagenesis, in which germline variants in humans with DLBCL have been identified by positional cloning, targeted sequencing or whole-exome/genome sequencing are depicted in Table 1. These variants are highly enriched in DLBCL patients in comparison with the control population from the ExAC database. The functional effects of missense mutations in these genes are not always understood. Of note, synonymous mutations can also be functionally relevant by affecting gene splicing.^15^

## DNA repair

Thirty-five genes of which germline variants have been observed in DLBCL are involved in DNA repair. Systematic studies of these germline mutations have thus far been very limited. A study using targeted capture sequencing of 73 key DNA repair genes reported novel and/or rare germline variants in these genes in 20 out of 22 DLBCL patients with an average of two but up to four variants (in four different genes) per patient.^16^ Although many of these missense mutations were unknown variants requiring functional validation, the authors showed that potentially functional germline mutations in mismatch repair genes were present in 27% of their DLBCL patients. These MMR mutations were associated with higher numbers of somatic mutations, which may serve as targets to the immune system and contribute to the sensitivity of such lymphomas to anti-PD1 immune checkpoint blockade.^3^

A DLBCL with microsatellite instability was reported in a colorectal cancer patient with a germline *MLH1* mutation.^17^ Although DLBCL may not be prominent in Lynch syndrome,^18^ hematological malignancies including DLBCL have a prevalence of 15%^19^ in constitutional mismatch repair deficiency resulting from bi-allelic germline mutations in one of the four MMR genes *MLH1*, *MSH2*, *MSH6* or *PMS2*.^20^

Several DNA repair genes are associated with hereditary cancer syndromes of which DLBCL is not considered a recurrent feature. DLBCL is not among the classical tumors in the Li–Fraumeni syndrome (LFS) caused by germline *TP53* mutations.^21^ LFS families do harbor lymphomas, and a possible LFS patient with a germline *TP53* mutation with a brain tumor and a subsequent DLBCL was reported from Japan.^22^ A single case report was published on the occurrence of breast cancer, ovarian cancer and DLBCL in a patient with a *BRCA1* mutation.^23^

Some of the germline mutations in the DNA damage response gene *CHEK2* observed in an analysis of 235 DLBCL patients^16^ are identical to those that were shown to confer an increased risk for solid tumors,^24^ and possibly essential thrombocythemia^25^ and polycythemia vera.^26^ Mechanistically, the *CHEK2* c.444+1G>A mutation leads to a truncated protein lacking the kinase activation domain and part of the functionally relevant forkhead homology-associated domain. The I157T mutation disturbs the interaction of the CHEK2 protein with p53 and BRCA1 in a dominant-negative manner.^27^

Targeted *CHEK2* gene sequence analysis in another group of 340 patients from the Czech republic with different types of B-cell lymphoma revealed many germline variants of the *CHEK2* gene including the well-known c.1100delC founder mutation. Nonsense- and missense-inherited *CHEK2* mutations were detected in 6.1% of 180 DLBCL cases.^28^ These additional mutations were not included in Table 1, because detailed information on the distribution of the variants was not provided specifically for DLBCL.

In general, the coincidence of DLBCL in patients with solid tumors is in all likelihood underreported, and the incidence of underlying germline DNA repair mutations is underestimated due to a lack of systematic DNA analyses.

## Immunodeficiency

For mutations in *LIG4* (DNA ligase IV),^29, 30, 31^ *NHEJ1* (XLF),^32^ *DCLRE1C* (Artemis),^33^ *NBN* (Nibrin),^34^ *WAS*,^35^ *PIK3CD*,^36^ *PIK3R1*,^37^ *SH2D1A*(SAP),^38, 39^ *IFNGR1*,^40^ *STAT3*,^41^ perforin-encoding *PRF1*^(refs 42, 43, 44)^ and *FAS*,^42, 45, 46^ an association with DLBCL has been reported. Severe combined immunodeficiencies due to homozygous or compound heterozygous mutations in *LIG4*, *NHEJ1* and *DCLRE1C*, genes involved in NHEJ, are known to cause EBV-associated DLBCLs, that become manifest in childhood. Heterozygous mutations, in for instance *LIG4*, are probably more prevalent than previously thought. Patients of over 40 years of age have been diagnosed with compound heterozygosity for a *LIG4* mutation, which can cause myelodysplasia.^47^ An adult patient with a homozygous congenital DNA ligase 4 mutation and non-EBV-associated DLBCL has also been reported.^30^ A 27-year-old woman with bilateral breast cancer and myelodysplasia was compound heterozygous for both an intronic mutation and a functional polymorphism of *DCLRE1C*.^48^ This illustrates that patients with inherited mutations in *LIG4* and *DCLRE1C* are not solely encountered by pediatric hematologists. In addition to immunodeficiency and increased propensity to develop lymphomas and other malignancies, patients with defects in NHEJ can exhibit developmental delay (short stature, microcephaly).^47^ A germline heterozygous splice site mutation in *PIK3R1* leads, like *PIK3CD* mutations, to an activated PI3Kδ syndrome (APDS) designated PASLI, which stands for p110δ-activating mutations causing senescent T cells, lymphadenopathy and immunodeficiency.^49, 50^ Depending on the underlying genetic defect, PASLI-CD/APDS1 and PASLI-R1/APDS2 are discerned. Both cause a combined immunodeficiency with phenotypic variability. Apart from infectious complications, lymphoproliferation, splenomegaly and autoimmunity, growth retardation and mild neurodevelopmental delay are among the potential clinical manifestations of inherited mutations in the *PIK3R1* gene. Age of onset of lymphoma (DLBCL, Hodgkin lymphoma, marginal zone lymphoma and Chronic Lymphocytic Leukemia) for APDS1 can range from 6 to 40 years.^37^ Size constraints prevented us from including in Table 1 all germline *SH2D1A* mutations that underlie X-linked lymphoproliferative syndrome type-I and can give rise to EBV-dependent and -independent DLBCL and Burkitt lymphomas in children and also in adults.^38, 39^ EBV-associated DLBCL has been reported in an adult with an inherited homozygous mutation in the *IFNGR1* gene.^40^ A second EBV-negative DLBCL (E van de Vosse, personal communication) was observed in a Dutch adult with a different homozygous mutation.^51^ Both mutations lead to a complete deficiency of the interferon gamma receptor 1 protein.^40, 51^

Like what has been observed with germline *FAS* mutations with^42^ or without^45^ a concomitant *PRF1* mutation, patients with the autoimmune lymphoproliferative syndrome caused by germline *STAT3*-activating mutations^52^ are expected to be at risk for developing DLBCL. So far, however, DLBCL has only been described as a consequence of a hyper IgE syndrome caused by a germline *STAT3*-inactivating mutation.^41^

## Transcription/chromatin remodeling

A single putative germline *TET2* mutation was seen in human DLBCL.^53^ The importance of a *KMT2A* alteration in DLBCL pathogenesis was underpinned by its segregation with the disease in a family.^54^ A germline heterozygous mutation in *TP63* was detected in a Japanese girl with ECC (ectrodactyly, ectodermal dysplasia, clefting) syndrome type 3, who subsequently developed DLBCL.^55^

## Signal transduction

Of the remaining genes presented in Table 1 two (*RNF31* and *TNFRSF13C*) may be classified as involved in signal transduction. Germline variants of the linear ubiquitin chain assembly complex subunit RNF31 provide an example how novel possibilities for therapeutic interventions may arise from the mechanistic understanding of the role of these proteins in a physiological and malignant setting. These variants are characterized as the gain-of-function polymorphisms that enhance the activity of the nuclear factor kappa B (NFkB) pathway.^56^ The germline H159Y mutation in TNFRSF13C may also lead, via TRAF3- and TRAF6-mediated signal transduction, to increased NFkB activity and appears to be associated with various lymphoma types including DLBCL.^57^

## Autophagy

Autophagy-associated *ULK4*^(ref. 58)^ gene contained a rare mutation in germline DNA from a DLBCL patient.^4^ Another variant of *ULK4* was shown to predispose to multiple myeloma and monoclonal gammopathy of undetermined significance.^59^ Whether Unc-51-like kinase 4 mutations also promote the development of DLBCL is by no means established yet.

## Chromosomal aberrations

Apart from single-nucleotide variants, and small insertions and deletions, germline alterations also encompass relatively rare fusion genes generated by gene duplication or inversion.^8^ Furthermore, germline copy-number neutral loss of heterozygosity involving chromosomes 3, 6, 7, 8, 9 and 20 was recently reported.^60^ Such germline loss of heterozygosity involved 29 regions of the genome with each loss occurring in at least 5% of DLBCL cases (*n*=40) versus 0–0.8% of normal controls (*n*=500). The functional oncogenic consequences of these chromosomal alterations remain to be elucidated.

## Germline mutations of somatically mutated genes in DLBCL

On the basis of gene expression analysis, DLBCL can be classified in two major subtypes of germinal center type and activated B-cell type.^61^ Although different somatic mutations are associated with these two DLBCL subtypes, these mutations are not strictly subtype-specific,^7^ and intra-tumoral heterogeneity may occur.^62^ Germinal center-type DLBCL are associated with mutations in *EZH2*, *GNA13*, *S1PR2*, *P2RY8*, *PTEN* and *ARHGEF1*, primarily affecting histone modification and cellular homing, respectively.^4, 63^ Activated B-cell type DLBCL may carry mutations in *CARD11* also known as *CARMA1*, *BCL10*, *MALT1*, *CD79A*, *CD79B*, *MYD88*, *TNFAIP3*, *SYK*, *PI3K*, *BTK* and *PKCβ*. These mutations mainly involve B-cell receptor signaling and the NFkB pathway.^64^

In addition to the 42 most frequently mutated genes,^4^ data were compiled from five studies^5, 6, 7, 8, 13^ to obtain a list of 626 genes in which somatic mutations have been found in human DLBCL (Supplementary Table S1). Of these 626 genes, 118 (depicted bold) are significantly mutated in DLBCL as compared to other tumors.^4, 5, 7^ In 24 (depicted in red) of the 626 genes, potential pathogenic mutations have also been observed in the germline of patients with DLBCL. These genes are presented in Table 1. Among the remaining 602 genes with somatic mutations in DLBCL, we identified 211 genes (depicted in blue) with mutations in germline DNA that may underlie other diseases, metabolic problems or developmental defects (Supplementary Information). These germline mutations, if not associated with embryonic lethality, represent candidate predisposing genes for the development of DLBCL.

## Genes associated with malignancy

By and large, the list of 211 genes somatically mutated in DLBCL, of which an inherited or a *de novo* mutation not known to predispose to this type of lymphoma was found, contains 39 genes of which germline alterations are known to predispose to cancer. Germline mutations in genes such as *POLE*, *WIF1* and *PTEN* have thus far been associated primarily with solid tumors.^65, 66, 67^ Inherited *MSH2* mutations also predominantly predispose for solid tumors, but a germline *MSH2* mutation was observed in a patient with follicular lymphoma.^16^ Heterozygous germline mutations in the histone methyltransferase *EZH2* gene cause the so-called Weaver syndrome with increased body height as one of its salient features. This syndrome may underlie lymphoblastic lymphoma, Acute Lymphoblastic Leukemia and Acute Myeloid Leukemia.^68, 69^ It is associated with intellectual disability of highly variable severity. These congenital mutations overlap with the somatic mutations in *EZH2* observed in hematological malignancies.

The finding of somatic mutations in *ETV6* in DLBCL is noteworthy as germline alterations of this transcription factor were shown to cause autosomal dominant transmission of thrombocytopenia, and predisposition to diverse hematological malignancies, colon cancer, melanoma, myopathy and gastrointestinal dysmotility.^70^ Germline mutations in *ETV6* occur in ~1% of children with Acute Lymphoblastic Leukemia.^71^

A recent study of somatic mutations in relapsed and refractory DLBCL added *NFkBIZ* as a new mutation target.^72^ Similar to some of the genes already discussed here, an inherited mutation in this gene may predispose to colorectal cancer.^73^

## Genes affecting immune function

Congenitally mutated *CARD11* has been associated with polyclonal B-cell lymphocytosis.^74, 75^ In this rare disorder, an identical mutation in *CARD11* as observed somatically in DLBCL leads to the so-called BENTA disease (B-cell expansion with NFkB and T-cell anergy), a congenital syndrome of lymphocytosis, splenomegaly, lymphadenopathy and T-cell anergy, which may evolve into a B-cell malignancy.^74, 75^ In contrast to these heterozygous gain-of-function mutations, homozygous loss-of-function mutations in *CARD11* compromise canonical NFkB signaling causing a combined immunodeficiency of variable severity with normal T- and B-cell numbers.^76^ The T-cell defect in this disorder may even be alleviated by an acquired somatic *CARD11* mutation.^77^

Loss-of-function mutations of the NFkB inhibitor NFKBIA will enhance NFkB activity. A heterozygous gain-of-function mutation in *NFKBIA* was shown to impair NFkB activation and associate with X-linked anhidrotic ectodermal dysplasia, T-cell immunodeficiency and polyclonal lymphocytosis.^78^ Congenital *CXCR4* and *BTK* mutations underlie primary immunodeficiency states WHIM (warts, hypogammaglobulinemia, infections and myelokathexis syndrome) and Bruton's type agammaglobulinemia.^79, 80^

Homozygosity or compound heterozygous mutations in *CERC1*, encoding adenosine deaminase 2, may cause hypogammaglobulinemia and polyarteritis nodosa,^81^ an autoimmune disorder associated with an increased incidence of lymphoma.^82^

Germline *BCL10*,^83^ *DOCK2*^(ref.84)^ and *RHOH*^85^ mutations cause a form of combined immunodeficiency by affecting B and T cells as well as other cells. By impairing anti-viral immunity, they can promote the development of lymphoma. A patient born with a RHOH deficiency indeed developed a Burkitt lymphoma.^85^ Inherited mutations in *CD79A*^86^ or *CD79B*^87^ can manifest themselves as hypogammaglobulinemia. *IRF8* mutations cause a dendritic cell deficiency in either an autosomal recessive or (phenotypically milder) autosomal dominant inheritance pattern.^88^ *CIITA* is a relatively frequent target of somatic mutations in DLBCL. Germline mutation of this gene in DLBCL was thus far only reported in canine DLBCL.^89^

## Genes with less obvious causality

One can readily imagine that alterations in genes that predispose to cancer in general or control immune function can play a causal role in DLBCL pathogenesis. Numerous genes that are recurrently mutated in DLBCL lack such intuitive function, and the possible contribution of their congenitally observed mutations to lymphomagenesis is much less obvious. Some of these genes may be solely somatically mutated in DLBCL as a consequence of their presence in late replicating regions, but this hypothesis awaits appropriate functional testing.^90^ When occurring in the germ line, some mutations can give rise to developmental, neurological, cardiac or metabolic disturbances. The precise nature of these mutations may differ from those observed somatically in DLBCL. The fact that mutations in these genes can occur in germline DNA does not automatically turn them into predisposition genes for DLBCL. In addition, phenotypic manifestations of these mutations can depend on homozygous or compound heterozygous alterations, which may or may not occur somatically. Versatility in terms of function of these genes can be developmental stage, cellular context, dosage, isoforms or alternative transcripts, and post-translational modifications dependent. Finally, germline variants of somatically mutated genes in DLBCL may constitute only weak predisposition genes on their own. As one variant can interact with a variant in another gene, a combination of variants could lead to a higher susceptibility for DLBCL. *WIF1* and *HNRNPA0* provide an example of such a combinatorial effect in solid tumors.^66^ For lymphomas, such an epistatic interaction was suggested for variants in the DNA repair genes *MRE11A* and *NBS1*.^91^

Large-scale whole-genome sequencing of germline DNA from patients diagnosed with DLBCL across different ethnic groups and geographic regions should reveal the true incidence and recurrent nature of underlying germline mutations. With only six matched tumor and normal DNA pairs, Pasqualucci *et al.*^13^ already identified by whole-exome sequencing 106 rare germline variants among which the *SERPINA6* gene. This gene is involved in corticosteroid bioavailability by encoding corticosteroid-binding globulin^92^ and it is also targeted by somatic mutations in DLBCL (Supplementary Table s1). Many of the germline variants described by Pasqualucci *et al.* belong to the same functional classes of genes that are somatically mutated in this disease and could well be relevant for its development. *MTMR15* now known as *FAN1* (Fanconi anemia-associated nuclease 1) of which germline mutations cause hereditary colorectal cancer and other solid tumors^93^ is an example of such a germline variant. Recognition of genes as bona fide DLBCL predisposing genes will require functional studies, animal models and segregation with disease (not exclusively DLBCL) in families.

## Phenotypic categorization of germline mutations of somatically mutated genes in DLBCL

Recurrent somatic mutations in DLBCL have been functionally categorized.^4, 5, 6, 7, 13^ As clinical symptoms caused by germline mutations in a gene can vary depending on the mutation and the patient, a strict phenotypic categorization is virtually impossible. In addition to the recurrent themes of tumor development and immune function as specified above, the clinical phenotypic spectrum of a given germline mutation frequently comprises complex syndromes that are difficult to assign to a single category. *NF1*, *KRAS*, *BRAF* and *A2ML1* mutations, for instance, underlie the so-called RASopathies and may give rise to several syndromes, that is, neurofibromatosis type I, cardiofaciocutaneous syndrome and Noonan syndrome.^94^ The large phenotypic spectrum of mutated genes is explained for some by their functional role as transcription factors. For example, *HNF1B* can predispose to solid tumors as well as maturity onset diabetes of the young.^95^

**Figure 1:**
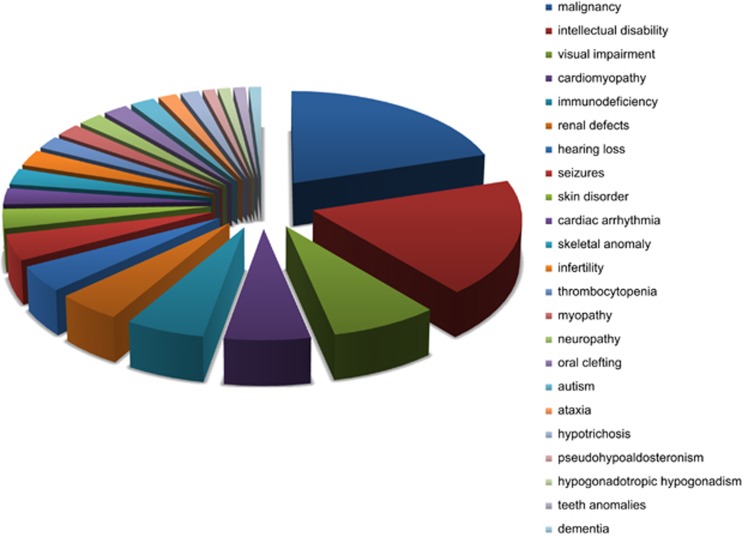
Phenotypic distribution germline mutations of genes somatically mutated in DLBCL.

Through compilation of the phenotypic consequences of germline mutations in genes not known to cause DLBCL of which somatic mutations have been observed in this lymphoma, certain prevalent phenotypes became evident. Bearing the limitations outlined above in mind, genes were assigned to a single phenotypic category. Phenotypes to which at least two genes could be linked are graphically displayed in Figure 1 and comprise 193 of the 211 genes somatically mutated in DLBCL of which a germline variant with a phenotype is known (Supplementary Information). In general, these variants can be considered pathogenic mutations and involve any part/organ of the human body. Perhaps most striking is the high number of genes of which inherited mutations are associated with intellectual disability.

## Non-coding genes

In addition to inherited or somatically acquired mutations in protein-coding genes, somatic mutations have been observed in DLBCL in genes encoding microRNAs^96^ and long non-coding RNAs.^97^ The B-Raf pseudogene *BRAFP1* represents an example of a non-coding transcript that can act as a competitive endogenous RNA to promote the development of DLBCL or other malignancies via the sequestration of microRNAs.^98^ Germline mutations in the long non-coding RNA *RMRP* cause cartilage–hair hypoplasia, immunodeficiency and an increased risk of malignancies including lymphomas (to the best of our knowledge no DLBCL has yet been reported). Two microRNAs derived from this long non-coding RNA cause gene silencing, which explains at least part of the effects of these *RMRP* mutations. The carrier frequency of these mutations is particularly high in the Amish and the Finnish populations.^99^

Aberrant somatic hypermutation of non-immunoglobulin genes by AID is an important mechanism for somatic oncogenic mutations in DLBCL. As RNAs guide AID to its site of activity,^10^ it is conceivable that germline mutations in these RNAs can misguide this enzyme and as such contribute to lymphomagenesis.

Epistatic interactions in DLBCL may occur (just like in solid tumors)^100^ not only between coding genes but also between coding and non-coding genes as well.

## Concluding remarks

The rapidly expanding knowledge on the somatic mutations in DLBCL has reinforced the notion of the heterogeneity of this disease. Molecularly targeted treatment strategies are currently being developed and may replace or be added to the established therapy backbone of R-CHOP immunochemotherapy. The activated B-cell subtype of DLBCL is the forerunner to explore pathway-directed therapeutic benefit.

Germline mutations add another layer of complexity. With this review, we aim at heighten physicians' awareness to the fact that inherited mutations that contribute to the pathogenesis of DLBCL can also predispose to other malignancies, various degrees of immunodeficiencies and a wide spectrum of organic disturbances of various severities.

A better understanding of DLBCL predisposition genes is clinically relevant, as their detection can lead to preventive measures in both patients and their relatives. Underlying defects in mismatch repair for instance can predispose to other cancers, which can be prevented by active surveillance, for example, colonoscopy. Increasing knowledge on the mode of action of these genes can also create new therapeutic options or influence the choice among established therapies. Inherited mutations in DNA double-strand break repair genes can be exploited to the benefit of the patient by selecting platinum-containing chemotherapy or the use of Poly ADP ribose polymerase inhibitors to induce synthetic lethality. Depending on the severity of the phenotype associated with the mutation, pre-implantation or prenatal diagnosis can be discussed, and upon further refinement of gene-editing technology even gene therapy can be envisioned.

## Figures and Tables

**Table 1:** Germline mutations in human DLBCL

*Gene*	*Sequence mutation*				*Reference*	*Allele frequency*	
	*Protein*	*c.DNA*	*chr*	*g.DNA*		*DLBCL*	*Controls*
*TP53*	V31I		17:	7579705C>T	16	0.05	0.0003
	C242Y		17:	7577555G>A	22	ND	NR
*TP53BP1*	T519A		15:		16	0.09	NR
	A1714S		15:			0.05	NR
*ATM*	R924W		11:	108139269G>A	16	0.05	0.00005
*RAD50*	L1125F		5:		16	0.05	NR
*RAD51B*	E346D				16	0.05	NR
	I357V		14:	68187283A>G	4	0.08	NR
*RAD54B*	P55L		8:	95470636G>A	16	0.05	0.00004
	I778V		8:	95390591T>C		0.05	0.0004
*FANCA*	T620I		16:	89842191G>A	16	0.05	0.00002
	R1321H		16:	89805934C>T		0.05	0.00005
*FANCG*	M431R		9:	35075603A>C	16	0.05	0.00004
*BLM*	S580P				16	0.05	NR
	V765I		15:	91310239G>A		0.05	0.0003
*BRCA1*	F93fs		17:		23	ND	NR
	Y856H			41244982A>G	16	0.05	0.002
*BRCA2*	C315S		13:	32906558T>A	16	0.05	0.0004
	S1744I		13:	32913723G>T		0.05	0.00002
	I1929V		13:	32914277A>G		0.05	0.001
*XRCC1*	A121T		19:	44058851C>T	16	0.05	0.0003
*XRCC5*	S349Y	c.1046C>A	2:		16	0.02	NR
*DDB1*	Y517C		11:	61081819T>C	16	0.05	0.0003
*MDC1*	H35L		6:	30682849T>A	16	0.05	0.00008
*CHEK2*	R3W	c.7C>T		29130703G>A		0.06	0.0002
	I157T	c.470T>C				0.02	NR
	H371Y	c.1111C>T	22:		16	0.03	NR
	E528K	c.1582G>A				0.02	NR
	E149Kfs*12	c.444+1G>A				0.006	NR
*PARP1*	S776G	c.2326A>G	1:		16	0.05	NR
*MLH1*	NR	NR	3:		17	ND	NR
*MLH3*	C40Y	c.119G>A	14:	75516240C>T	16	0.05	0.00002
	S946F	c.2837C>T				0.05	NR
	I988M	c.2964C>G	14:	75513395G>C		0.05	0.00006
	L1111F	c.3331C>T	14:	75509130G>A		0.05	0.000008
*MSH3*	P657S		5:	80063824C>T	16	0.05	0.00003
	R1061G		5:	80168985A>G		0.09	0.0002
*MSH6*	T563N		2:		16	0.05	NR
*PMS1*	L369P				16	0.05	NR
	D397E		2:	190719189T>G		0.05	0.00002
*PMS2*	S128L		7:	6042238G>A	16	0.05	0.0008
	M362K					0.05	NR
*POLB*	R333Q		8:	42229165G>A	16	0.05	0.00003
*DNTT*	V37I		10:	98064363G>A	16	0.05	0.0008
	R335W		10:	98087353C>T		0.05	0.00005
*PRKDC*	D566N		8:	48845660C>T	16	0.05	0.00002
	K1984N					0.05	NR
*RPA1*	G160R		17:	1778978G>C	16	0.05	0.00006
*TNFAIP3*	P714S		6:	138202223C>T	16	0.05	0.0002
*UNG*	E121Q		12:		16	0.05	NR
*LIG1*	T311M		19:	48643383G>A	16	0.05	0.00002
*LIG3*	R343Q		17:	33318120G>A	16	0.05	0.00007
	M249V	c.745A>G	13:		29	ND	NR
		c.1270_1274delAAAAG			3030	ND	NR
*LIG4*	R278H	c.833G>A			30	ND	NR
		c.2736+3delC			31	ND	NR
*NHEJ1*	R178X	c.622C>T	2:		32	ND	NR
*DCLRE1C*	D451fs	c.1384_1390del			33	ND	NR
			10:	del Exon 1-3			
*NBN*		c.657_661delACAAA	8:		34	ND	NR
*WAS*			X:	41delG	35	ND	NR
*PIK3CD*	D911E;D935I		1:	9706935T>A	4	0.08	NR
	E1021K				36	ND	NR
*PIK3R1*	del434-475		5:	67589663G>A	37	ND	NR
				67589663G>C		ND	NR
				67589663G>T		ND	NR
				67589662G>C		ND	NR
				67589664T>A		ND	NR
				67589664delTG		ND	NR
				67589664T>G		ND	NR
*SH2D1A*	W64*		X:	123400664G>A	38	ND	0.00001
*IFNGR1*	V10Sfs*5	c.25del^a^	6:	22delC	39	ND	NR
	V68Kfs*6	c.373+1G>T		137527272C>A	50	ND	0.000008
*STAT3*	K340T	c.1259A>C	17:		40	ND	NR
	R382Q	c.1145G>A				ND	NR
	E690P699del	c.2069del30bp				ND	NR
*PRF1*	A3A	c.9C>T			43	0.03	NR
	A109G	c.326C>G				0.03	NR
	F169S	c.506T>C				0.03	NR
	N252S	c.755A>G	10:	72358722T>C	41	ND	0.005
	R385W	c.1153C>T	10:	72358324G>A	43	0.03	0.002
	T435M	c.1304C>T	10:	72358173G>A	42	ND	0.00002
	T450M	c.1349C>T	10:	72358128G>A	42	ND	0.00005
*FAS*	T225P	c.915A>C	10:		44	ND	NR
	D244V	c.973A>T			45	ND	NR
				IVS7nt1A>G	41	ND	NR
*TP63*	N312G		3:		54	ND	NR
*TET2*^b^		c.3473–1G>A	4:		52	0.01	NR
*KMT2A*	H1845N	c.5533C>A	11:		53	ND	NR
*RNF31*	Q584H		14:	24620708G>T	55	0.02	0.001
	Q622L		14:	24620821A>T		0.06	0.003
*TNFRSF13C*	H159Y		22:	42321451G>A	56	0.05	0.006
*ULK4*	S770R		3:	41770870T>G	4	0.08	NR

Abbreviations: DLBCL, diffuse large B-cell lymphoma; ND, not determined; NR, not reported; NA, not applicable.

Allele frequency in DLBCL calculated from literature.^4, 16, 43, 52, 55, 56^ Data of controls are from the Exome Aggregation Consortium (ExAC), Cambridge, MA, USA (http://exac.broadinstitute.org).

a Exon 1 of the *IFNGR1* gene harbors four cytosines; c.del25 and 22delC refer to the same mutation.

b Mutation of which germline nature is likely.

## References

[bib1] Li Y, Wang Y, Wang Z, Yi D, Ma S. Racial differences in three major NHL subtypes: descriptive epidemiology. Cancer Epidemiol 2015; 39: 8–13. 25560974 10.1016/j.canep.2014.12.001 PMC4323749

[bib2] Tilly H, Gomes da Silva M, Vitolo U, Jack A, Meignan M, Lopez-Guillermo A et al. Diffuse large B-cell lymphoma (DLBCL): ESMO Clinical Practice Guidelines for diagnosis, treatment and follow-up. Ann Oncol 2015; 26(Suppl 5): v116–v125. 26314773 10.1093/annonc/mdv304

[bib3] Georgiou K, Chen L, Berglund M, Ren W, de Miranda NF, Lisboa S et al. Genetic basis of PD-L1 overexpression in diffuse large B-cell lymphomas. Blood 2016; 127: 3026–3034. 27030389 10.1182/blood-2015-12-686550

[bib4] Morin RD, Mendez-Lago M, Mungall AJ, Goya R, Mungall KL, Corbett RD et al. Frequent mutation of histone-modifying genes in non-Hodgkin lymphoma. Nature 2011; 476: 298–303. 21796119 10.1038/nature10351 PMC3210554

[bib5] Lohr JG, Stojanov P, Lawrence MS, Auclair D, Chapuy B, Sougnez C et al. Discovery and prioritization of somatic mutations in diffuse large B-cell lymphoma (DLBCL) by whole-exome sequencing. Proc Natl Acad Sci USA 2012; 109: 3879–3884. 22343534 10.1073/pnas.1121343109 PMC3309757

[bib6] Zhang J, Grubor V, Love CL, Banerjee A, Richards KL, Mieczkowski PA et al. Genetic heterogeneity of diffuse large B-cell lymphoma. Proc Natl Acad Sci USA 2013; 110: 1398–1403. 23292937 10.1073/pnas.1205299110 PMC3557051

[bib7] de Miranda NF, Georgiou K, Chen L, Wu C, Gao Z, Zaravinos A et al. Exome sequencing reveals novel mutation targets in diffuse large B-cell lymphomas derived from Chinese patients. Blood 2014; 124: 2544–2553. 25171927 10.1182/blood-2013-12-546309 PMC4199956

[bib8] Morin RD, Mungall K, Pleasance E, Mungall AJ, Goya R, Huff RD et al. Mutational and structural analysis of diffuse large B-cell lymphoma using whole-genome sequencing. Blood 2013; 122: 1256–1265. 23699601 10.1182/blood-2013-02-483727 PMC3744992

[bib9] Qian J, Wang Q, Dose M, Pruett N, Kieffer-Kwon KR, Resch W et al. B cell super-enhancers and regulatory clusters recruit AID tumorigenic activity. Cell 2014; 159: 1524–1537. 25483777 10.1016/j.cell.2014.11.013 PMC4272762

[bib10] Zheng S, Vuong BQ, Vaidyanathan B, Lin JY, Huang FT, Chaudhuri J, Non-coding RNA. Generated following Lariat Debranching Mediates Targeting of AID to DNA. Cell 2015; 161: 762–773. 25957684 10.1016/j.cell.2015.03.020 PMC4426339

[bib11] Robbiani DF, Deroubaix S, Feldhahn N, Oliveira TY, Callen E, Wang Q et al. Plasmodium infection promotes genomic instability and AID-dependent B cell lymphoma. Cell 2015; 162: 727–737. 26276629 10.1016/j.cell.2015.07.019 PMC4538708

[bib12] Tan J, Pieper K, Piccoli L, Abdi A, Foglierini M, Geiger R et al. A LAIR1 insertion generates broadly reactive antibodies against malaria variant antigens. Nature 2016; 529: 105–109. 26700814 10.1038/nature16450 PMC4869849

[bib13] Pasqualucci L, Trifonov V, Fabbri G, Ma J, Rossi D, Chiarenza A et al. Analysis of the coding genome of diffuse large B-cell lymphoma. Nat Genet 2011; 43: 830–837. 21804550 10.1038/ng.892 PMC3297422

[bib14] Stephens PJ, Greenman CD, Fu B, Yang F, Bignell GR, Mudie LJ et al. Massive genomic rearrangement acquired in a single catastrophic event during cancer development. Cell 2011; 144: 27–40. 21215367 10.1016/j.cell.2010.11.055 PMC3065307

[bib15] Supek F, Minana B, Valcarcel J, Gabaldon T, Lehner B. Synonymous mutations frequently act as driver mutations in human cancers. Cell 2014; 156: 1324–1335. 24630730 10.1016/j.cell.2014.01.051

[bib16] de Miranda NF, Peng R, Georgiou K, Wu C, Falk Sorqvist E, Berglund M et al. DNA repair genes are selectively mutated in diffuse large B cell lymphomas. J Exp Med 2013; 210: 1729–1742. 23960188 10.1084/jem.20122842 PMC3754869

[bib17] Cheah CY, Dsouza L, Taggart MW, Schlette EJ, Turturro F. Diffuse large B-cell lymphoma with microsatellite instability developing in the setting of Muir-Torre variant hereditary non-polyposis colon cancer. J Clin Pathol 2015; 68: 755–757. 26008772 10.1136/jclinpath-2015-203039

[bib18] Lynch HT, Snyder CL, Shaw TG, Heinen CD, Hitchins MP. Milestones of Lynch syndrome: 1895-2015. Nat Rev Cancer 2015; 15: 181–194. 25673086 10.1038/nrc3878

[bib19] Bakry D, Aronson M, Durno C, Rimawi H, Farah R, Alharbi QK et al. Genetic and clinical determinants of constitutional mismatch repair deficiency syndrome: report from the constitutional mismatch repair deficiency consortium. Eur J Cancer 2014; 50: 987–996. 24440087 10.1016/j.ejca.2013.12.005

[bib20] Wimmer K, Kratz CP, Vasen HF, Caron O, Colas C, Entz-Werle N et al. Diagnostic criteria for constitutional mismatch repair deficiency syndrome: suggestions of the European consortium 'care for CMMRD' (C4CMMRD). J Med Genet 2014; 51: 355–365. 24737826 10.1136/jmedgenet-2014-102284

[bib21] Bougeard G, Renaux-Petel M, Flaman JM, Charbonnier C, Fermey P, Belotti M et al. Revisiting Li-Fraumeni syndrome from TP53 mutation carriers. J Clin Oncol 2015; 33: 2345–2352. 26014290 10.1200/JCO.2014.59.5728

[bib22] Murakawa Y, Yokoyama A, Kato S, Yoshioka T, Ichinohasama R, Kumabe T et al. Astrocytoma and B-cell lymphoma development in a man with a p53 germline mutation. Jpn J Clin Oncol 1998; 28: 631–637. 9839505 10.1093/jjco/28.10.631

[bib23] Kim HS, Lee SW, Choi YJ, Shin SW, Kim YH, Cho MS et al. Novel germline mutation of BRCA1 gene in a 56-year-old woman with breast cancer, ovarian cancer, and diffuse large B-cell lymphoma. Cancer Res Treat 2015; 47: 534–538. 25483746 10.4143/crt.2013.151 PMC4506095

[bib24] Cybulski C, Gorski B, Huzarski T, Masojc B, Mierzejewski M, Debniak T et al. CHEK2 is a multiorgan cancer susceptibility gene. Am J Hum Genet 2004; 75: 1131–1135. 15492928 10.1086/426403 PMC1182149

[bib25] Janiszewska H, Bak A, Pilarska M, Heise M, Junkiert-Czarnecka A, Kuliszkiewicz-Janus M et al. A risk of essential thrombocythemia in carriers of constitutional CHEK2 gene mutations. Haematologica 2012; 97: 366–370. 22058216 10.3324/haematol.2011.049494 PMC3291590

[bib26] Janiszewska H, Bak A, Hartwig M, Kuliszkiewicz-Janus M, Calbecka M, Jazwiec B et al. The germline mutations of the CHEK2 gene are associated with an increased risk of polycythaemia vera. Br J Haematol 2016; 173: 150–152. 26084796 10.1111/bjh.13559

[bib27] Bartek J, Lukas J. Chk1 and Chk2 kinases in checkpoint control and cancer. Cancer Cell 2003; 3: 421–429. 12781359 10.1016/s1535-6108(03)00110-7

[bib28] Havranek O, Kleiblova P, Hojny J, Lhota F, Soucek P, Trneny M et al. Association of germline CHEK2 gene variants with risk and prognosis of non-Hodgkin lymphoma. PLoS ONE 2015; 10: e0140819. 26506619 10.1371/journal.pone.0140819 PMC4624763

[bib29] Toita N, Hatano N, Ono S, Yamada M, Kobayashi R, Kobayashi I et al. Epstein-Barr virus-associated B-cell lymphoma in a patient with DNA ligase IV (LIG4) syndrome. Am J Med Genet A 2007; 143A: 742–745. 17345618 10.1002/ajmg.a.31644

[bib30] Bacon CM, Wilkinson SJ, Spickett GP, Barge D, Lucraft HH, Jackson G et al. Epstein-Barr virus-independent diffuse large B-cell lymphoma in DNA ligase 4 deficiency. J Allergy Clin Immunol 2013; 131: 1237–1239, 1239 e1231. 23228243 10.1016/j.jaci.2012.10.027

[bib31] Sharapova SO, Chang EY, Guryanova IE, Proleskovskaya IV, Fedorova AS, Rutskaya EA et al. Next generation sequencing revealed DNA ligase IV deficiency in a "developmentally normal" patient with massive brain Epstein-Barr virus-positive diffuse large B-cell lymphoma. Clin Immunol 2016; 163: 108–110. 26774591 10.1016/j.clim.2016.01.002

[bib32] Patiroglu T, Akar HH, van der Burg M, Kontas O. A case of XLF deficiency presented with diffuse large B cell lymphoma in the brain. Clin Immunol 2015; 161: 394–395. 26119972 10.1016/j.clim.2015.06.009

[bib33] Moshous D, Pannetier C, Chasseval Rd R, Deist Fl F, Cavazzana-Calvo M, Romana S et al. Partial T and B lymphocyte immunodeficiency and predisposition to lymphoma in patients with hypomorphic mutations in Artemis. J Clin Invest 2003; 111: 381–387. 12569164 10.1172/JCI16774 PMC151863

[bib34] Dembowska-Baginska B, Perek D, Brozyna A, Wakulinska A, Olczak-Kowalczyk D, Gladkowska-Dura M et al. Non-Hodgkin lymphoma (NHL) in children with Nijmegen Breakage syndrome (NBS). Pediatr Blood Cancer 2009; 52: 186–190. 18937313 10.1002/pbc.21789

[bib35] Koga Y, Takada H, Suminoe A, Ohga S, Hara T. Successful treatment of non-Hodgkin's lymphoma using R-CHOP in a patient with Wiskott-Aldrich syndrome followed by a reduced-intensity stem cell transplant. Pediatr Transplant 2014; 18: E208–E211. 24930759 10.1111/petr.12297

[bib36] Crank MC, Grossman JK, Moir S, Pittaluga S, Buckner CM, Kardava L et al. Mutations in PIK3CD can cause hyper IgM syndrome (HIGM) associated with increased cancer susceptibility. J Clin Immunol 2014; 34: 272–276. 24610295 10.1007/s10875-014-0012-9 PMC4159085

[bib37] Elkaim E, Neven B, Bruneau J, Mitsui-Sekinaka K, Stanislas A, Heurtier L et al. Clinical and immunologic phenotype associated with activated phosphoinositide 3-kinase delta syndrome 2: a cohort study. J Allergy Clin Immunol 2016; 138: 210–218,e219. 27221134 10.1016/j.jaci.2016.03.022

[bib38] Hervier B, Latour S, Loussouarn D, Rimbert M, De-Saint-Basile G, Picard C et al. An atypical case of X-linked lymphoproliferative disease revealed as a late cerebral lymphoma. J Neuroimmunol 2010; 218: 125–128. 19906447 10.1016/j.jneuroim.2009.10.012

[bib39] Sandlund JT, Shurtleff SA, Onciu M, Horwitz E, Leung W, Howard V et al. Frequent mutations in SH2D1A (XLP) in males presenting with high-grade mature B-cell neoplasms. Pediatr Blood Cancer 2013; 60: E85–E87. 23589280 10.1002/pbc.24525 PMC4758190

[bib40] Bax HI, Freeman AF, Anderson VL, Vesterhus P, Laerum D, Pittaluga S et al. B-cell lymphoma in a patient with complete interferon gamma receptor 1 deficiency. J Clin Immunol 2013; 33: 1062–1066. 23800860 10.1007/s10875-013-9907-0 PMC3729015

[bib41] Kumanovics A, Perkins SL, Gilbert H, Cessna MH, Augustine NH, Hill HR. Diffuse large B cell lymphoma in hyper-IgE syndrome due to STAT3 mutation. J Clin Immunol 2010; 30: 886–893. 20859667 10.1007/s10875-010-9452-z

[bib42] Clementi R, Dagna L, Dianzani U, Dupre L, Dianzani I, Ponzoni M et al. Inherited perforin and Fas mutations in a patient with autoimmune lymphoproliferative syndrome and lymphoma. N Engl J Med 2004; 351: 1419–1424. 15459303 10.1056/NEJMoa041432

[bib43] Clementi R, Locatelli F, Dupre L, Garaventa A, Emmi L, Bregni M et al. A proportion of patients with lymphoma may harbor mutations of the perforin gene. Blood 2005; 105: 4424–4428. 15728124 10.1182/blood-2004-04-1477

[bib44] Ding Q, Yang LY. Perforin gene mutations in 77 Chinese patients with lymphomas. World J Emerg Med 2013; 4: 128–132. 25215106 10.5847/wjem.j.issn.1920-8642.2013.02.008 PMC4129835

[bib45] Straus SE, Jaffe ES, Puck JM, Dale JK, Elkon KB, Rosen-Wolff A et al. The development of lymphomas in families with autoimmune lymphoproliferative syndrome with germline Fas mutations and defective lymphocyte apoptosis. Blood 2001; 98: 194–200. 11418480 10.1182/blood.v98.1.194

[bib46] Infante AJ, Britton HA, DeNapoli T, Middelton LA, Lenardo MJ, Jackson CE et al. The clinical spectrum in a large kindred with autoimmune lymphoproliferative syndrome caused by a Fas mutation that impairs lymphocyte apoptosis. J Pediatr 1998; 133: 629–633. 9821419 10.1016/s0022-3476(98)70102-7

[bib47] O'Driscoll M, Cerosaletti KM, Girard PM, Dai Y, Stumm M, Kysela B et al. DNA ligase IV mutations identified in patients exhibiting developmental delay and immunodeficiency. Mol Cell 2001; 8: 1175–1185. 11779494 10.1016/s1097-2765(01)00408-7

[bib48] Woodbine L, Grigoriadou S, Goodarzi AA, Riballo E, Tape C, Oliver AW et al. An Artemis polymorphic variant reduces Artemis activity and confers cellular radiosensitivity. DNA Repair 2010; 9: 1003–1010. 20674517 10.1016/j.dnarep.2010.07.001

[bib49] Deau MC, Heurtier L, Frange P, Suarez F, Bole-Feysot C, Nitschke P et al. A human immunodeficiency caused by mutations in the PIK3R1 gene. J Clin Invest 2014; 124: 3923–3928. 25133428 10.1172/JCI75746 PMC4153704

[bib50] Lucas CL, Zhang Y, Venida A, Wang Y, Hughes J, McElwee J et al. Heterozygous splice mutation in PIK3R1 causes human immunodeficiency with lymphoproliferation due to dominant activation of PI3K. J Exp Med 2014; 211: 2537–2547. 25488983 10.1084/jem.20141759 PMC4267241

[bib51] de Vor IC, van der Meulen PM, Bekker V, Verhard EM, Breuning MH, Harnisch E et al. Deletion of the entire interferon-gamma receptor 1 gene causing complete deficiency in three related patients. J Clin Immunol 2016; 36: 195–203. 26931784 10.1007/s10875-016-0244-y PMC4792359

[bib52] Flanagan SE, Haapaniemi E, Russell MA, Caswell R, Lango Allen H, De Franco E et al. Activating germline mutations in STAT3 cause early-onset multi-organ autoimmune disease. Nat Genet 2014; 46: 812–814. 25038750 10.1038/ng.3040 PMC4129488

[bib53] Asmar F, Punj V, Christensen J, Pedersen MT, Pedersen A, Nielsen AB et al. Genome-wide profiling identifies a DNA methylation signature that associates with TET2 mutations in diffuse large B-cell lymphoma. Haematologica 2013; 98: 1912–1920. 23831920 10.3324/haematol.2013.088740 PMC3856967

[bib54] Saarinen S, Kaasinen E, Karjalainen-Lindsberg ML, Vesanen K, Aavikko M, Katainen R et al. Primary mediastinal large B-cell lymphoma segregating in a family: exome sequencing identifies MLL as a candidate predisposition gene. Blood 2013; 121: 3428–3430. 23457195 10.1182/blood-2012-06-437210

[bib55] Akahoshi K, Sakazume S, Kosaki K, Ohashi H, Fukushima Y. EEC syndrome type 3 with a heterozygous germline mutation in the P63 gene and B cell lymphoma. Am J Med Genet A 2003; 120A: 370–373. 12838557 10.1002/ajmg.a.20064

[bib56] Yang Y, Schmitz R, Mitala J, Whiting A, Xiao W, Ceribelli M et al. Essential role of the linear ubiquitin chain assembly complex in lymphoma revealed by rare germline polymorphisms. Cancer Discov 2014; 4: 480–493. 24491438 10.1158/2159-8290.CD-13-0915 PMC3992927

[bib57] Hildebrand JM, Luo Z, Manske MK, Price-Troska T, Ziesmer SC, Lin W et al. A BAFF-R mutation associated with non-Hodgkin lymphoma alters TRAF recruitment and reveals new insights into BAFF-R signaling. J Exp Med 2010; 207: 2569–2579. 21041452 10.1084/jem.20100857 PMC2989778

[bib58] Lebovitz CB, Robertson AG, Goya R, Jones SJ, Morin RD, Marra MA et al. Cross-cancer profiling of molecular alterations within the human autophagy interaction network. Autophagy 2015; 11: 1668–1687. 26208877 10.1080/15548627.2015.1067362 PMC4590660

[bib59] Weinhold N, Johnson DC, Rawstron AC, Forsti A, Doughty C, Vijayakrishnan J et al. Inherited genetic susceptibility to monoclonal gammopathy of unknown significance. Blood 2014; 123: 2513–2517. 24449210 10.1182/blood-2013-10-532283

[bib60] Sebastian E, Alcoceba M, Martin-Garcia D, Blanco O, Sanchez-Barba M, Balanzategui A et al. High-resolution copy number analysis of paired normal-tumor samples from diffuse large B cell lymphoma. Ann Hematol 2016; 95: 253–262. 26573278 10.1007/s00277-015-2552-3

[bib61] Alizadeh AA, Eisen MB, Davis RE, Ma C, Lossos IS, Rosenwald A et al. Distinct types of diffuse large B-cell lymphoma identified by gene expression profiling. Nature 2000; 403: 503–511. 10676951 10.1038/35000501

[bib62] Kusakabe M, Wang XH, Simkin G, Meskas J, Zhang C, Ennishi D et al. Mass cytometry based classification of inter- and intra-tumoral heterogeneity in diffuse large B-cell lymphoma. Blood 2015; 126: 3208.

[bib63] Muppidi JR, Schmitz R, Green JA, Xiao W, Larsen AB, Braun SE et al. Loss of signalling via Galpha13 in germinal centre B-cell-derived lymphoma. Nature 2014; 516: 254–258. 25274307 10.1038/nature13765 PMC4267955

[bib64] Davis RE, Ngo VN, Lenz G, Tolar P, Young RM, Romesser PB et al. Chronic active B-cell-receptor signalling in diffuse large B-cell lymphoma. Nature 2010; 463: 88–92. 20054396 10.1038/nature08638 PMC2845535

[bib65] Hansen MF, Johansen J, Bjornevoll I, Sylvander AE, Steinsbekk KS, Saetrom P et al. A novel POLE mutation associated with cancers of colon, pancreas, ovaries and small intestine. Fam Cancer 2015; 14: 437–448. 25860647 10.1007/s10689-015-9803-2 PMC4559173

[bib66] Wei C, Peng B, Han Y, Chen WV, Rother J, Tomlinson GE et al. Mutations of HNRNPA0 and WIF1 predispose members of a large family to multiple cancers. Fam Cancer 2015; 14: 297–306. 25716654 10.1007/s10689-014-9758-8 PMC4589301

[bib67] Tan MH, Mester JL, Ngeow J, Rybicki LA, Orloff MS, Eng C. Lifetime cancer risks in individuals with germline PTEN mutations. Clin Cancer Res 2012; 18: 400–407. 22252256 10.1158/1078-0432.CCR-11-2283 PMC3261579

[bib68] Tatton-Brown K, Hanks S, Ruark E, Zachariou A, Duarte Sdel V, Ramsay E et al. Germline mutations in the oncogene EZH2 cause Weaver syndrome and increased human height. Oncotarget 2011; 2: 1127–1133. 22190405 10.18632/oncotarget.385 PMC3282071

[bib69] Usemann J, Ernst T, Schafer V, Lehmberg K, Seeger K. EZH2 mutation in an adolescent with Weaver syndrome developing acute myeloid leukemia and secondary hemophagocytic lymphohistiocytosis. Am J Med Genet A 2016; 170: 1274–1277. 10.1002/ajmg.a.37562 26762561

[bib70] Zhang MY, Churpek JE, Keel SB, Walsh T, Lee MK, Loeb KR et al. Germline ETV6 mutations in familial thrombocytopenia and hematologic malignancy. Nat Genet 2015; 47: 180–185. 25581430 10.1038/ng.3177 PMC4540357

[bib71] Moriyama T, Metzger ML, Wu G, Nishii R, Qian M, Devidas M et al. Germline genetic variation in ETV6 and risk of childhood acute lymphoblastic leukaemia: a systematic genetic study. Lancet Oncol 2015; 16: 1659–1666. 26522332 10.1016/S1470-2045(15)00369-1 PMC4684709

[bib72] Morin RD, Assouline S, Alcaide M, Mohajeri A, Johnston RL, Chong L et al. Genetic Landscapes of Relapsed and Refractory Diffuse Large B-Cell Lymphomas. Clin Cancer Res 2016; 22: 2290–2300. 26647218 10.1158/1078-0432.CCR-15-2123

[bib73] Esteban-Jurado C, Vila-Casadesus M, Garre P, Lozano JJ, Pristoupilova A, Beltran S et al. Whole-exome sequencing identifies rare pathogenic variants in new predisposition genes for familial colorectal cancer. Genet Med 2015; 17: 131–142. 25058500 10.1038/gim.2014.89 PMC4318970

[bib74] Brohl AS, Stinson JR, Su HC, Badgett T, Jennings CD, Sukumar G et al. Germline CARD11 Mutation in a Patient with Severe Congenital B Cell Lymphocytosis. J Clin Immunol 2015; 35: 32–46. 25352053 10.1007/s10875-014-0106-4 PMC4466218

[bib75] Snow AL, Xiao W, Stinson JR, Lu W, Chaigne-Delalande B, Zheng L et al. Congenital B cell lymphocytosis explained by novel germline CARD11 mutations. J Exp Med 2012; 209: 2247–2261. 23129749 10.1084/jem.20120831 PMC3501355

[bib76] Stepensky P, Keller B, Buchta M, Kienzler AK, Elpeleg O, Somech R et al. Deficiency of caspase recruitment domain family, member 11 (CARD11), causes profound combined immunodeficiency in human subjects. J Allergy Clin Immunol 2013; 131: 477–485,e471. 23374270 10.1016/j.jaci.2012.11.050

[bib77] Fuchs S, Rensing-Ehl A, Pannicke U, Lorenz MR, Fisch P, Jeelall Y et al. Omenn syndrome associated with a functional reversion due to a somatic second-site mutation in CARD11 deficiency. Blood 2015; 126: 1658–1669. 26289640 10.1182/blood-2015-03-631374 PMC4654427

[bib78] Courtois G, Smahi A, Reichenbach J, Doffinger R, Cancrini C, Bonnet M et al. A hypermorphic IkappaBalpha mutation is associated with autosomal dominant anhidrotic ectodermal dysplasia and T cell immunodeficiency. J Clin Invest 2003; 112: 1108–1115. 14523047 10.1172/JCI18714 PMC198529

[bib79] Al Ustwani O, Kurzrock R, Wetzler M. Genetics on a WHIM. Br J Haematol 2014; 164: 15–23. 24111611 10.1111/bjh.12574 PMC3961560

[bib80] Mohamed AJ, Yu L, Backesjo CM, Vargas L, Faryal R, Aints A et al. Bruton's tyrosine kinase (Btk): function, regulation, and transformation with special emphasis on the PH domain. Immunol Rev 2009; 228: 58–73. 19290921 10.1111/j.1600-065X.2008.00741.x

[bib81] Navon Elkan P, Pierce SB, Segel R, Walsh T, Barash J, Padeh S et al. Mutant adenosine deaminase 2 in a polyarteritis nodosa vasculopathy. N Engl J Med 2014; 370: 921–931. 24552285 10.1056/NEJMoa1307362

[bib82] Fallah M, Liu X, Ji J, Forsti A, Sundquist K, Hemminki K. Autoimmune diseases associated with non-Hodgkin lymphoma: a nationwide cohort study. Ann Oncol 2014; 25: 2025–2030. 25081899 10.1093/annonc/mdu365

[bib83] Torres JM, Martinez-Barricarte R, Garcia-Gomez S, Mazariegos MS, Itan Y, Boisson B et al. Inherited BCL10 deficiency impairs hematopoietic and nonhematopoietic immunity. J Clin Invest 2014; 124: 5239–5248. 25365219 10.1172/JCI77493 PMC4348943

[bib84] Dobbs K, Dominguez Conde C, Zhang SY, Parolini S, Audry M, Chou J et al. Inherited DOCK2 deficiency in patients with early-onset invasive infections. N Engl J Med 2015; 372: 2409–2422. 26083206 10.1056/NEJMoa1413462 PMC4480434

[bib85] Crequer A, Troeger A, Patin E, Ma CS, Picard C, Pedergnana V et al. Human RHOH deficiency causes T cell defects and susceptibility to EV-HPV infections. J Clin Invest 2012; 122: 3239–3247. 22850876 10.1172/JCI62949 PMC3428089

[bib86] Minegishi Y, Coustan-Smith E, Rapalus L, Ersoy F, Campana D, Conley ME. Mutations in Igalpha (CD79a) result in a complete block in B-cell development. J Clin Invest 1999; 104: 1115–1121. 10525050 10.1172/JCI7696 PMC408581

[bib87] Dobbs AK, Yang T, Farmer D, Kager L, Parolini O, Conley ME. Cutting edge: a hypomorphic mutation in Igbeta (CD79b) in a patient with immunodeficiency and a leaky defect in B cell development. J Immunol 2007; 179: 2055–2059. 17675462 10.4049/jimmunol.179.4.2055

[bib88] Hambleton S, Salem S, Bustamante J, Bigley V, Boisson-Dupuis S, Azevedo J et al. IRF8 mutations and human dendritic-cell immunodeficiency. N Engl J Med 2011; 365: 127–138. 21524210 10.1056/NEJMoa1100066 PMC3136554

[bib89] Bushell KR, Kim Y, Chan FC, Ben-Neriah S, Jenks A, Alcaide M et al. Genetic inactivation of TRAF3 in canine and human B-cell lymphoma. Blood 2015; 125: 999–1005. 25468570 10.1182/blood-2014-10-602714

[bib90] Lawrence MS, Stojanov P, Polak P, Kryukov GV, Cibulskis K, Sivachenko A et al. Mutational heterogeneity in cancer and the search for new cancer-associated genes. Nature 2013; 499: 214–218. 23770567 10.1038/nature12213 PMC3919509

[bib91] Rendleman J, Antipin Y, Reva B, Adaniel C, Przybylo JA, Dutra-Clarke A et al. Genetic variation in DNA repair pathways and risk of non-Hodgkin's lymphoma. PloS ONE 2014; 9: e101685. 25010664 10.1371/journal.pone.0101685 PMC4092067

[bib92] Simard M, Hill LA, Lewis JG, Hammond GL. Naturally occurring mutations of human corticosteroid-binding globulin. J Clin Endocrinol Metab 2015; 100: E129–E139. 25322275 10.1210/jc.2014-3130

[bib93] Segui N, Mina LB, Lazaro C, Sanz-Pamplona R, Pons T, Navarro M et al. Germline mutations in FAN1 cause hereditary colorectal cancer by impairing DNA repair. Gastroenterology 2015; 149: 563–566. 26052075 10.1053/j.gastro.2015.05.056

[bib94] Aoki Y, Niihori T, Inoue S, Matsubara Y. Recent advances in RASopathies. J Hum Genet 2016; 61: 33–39. 26446362 10.1038/jhg.2015.114

[bib95] Rebouissou S, Vasiliu V, Thomas C, Bellanne-Chantelot C, Bui H, Chretien Y et al. Germline hepatocyte nuclear factor 1alpha and 1beta mutations in renal cell carcinomas. Hum Mol Genet 2005; 14: 603–614. 15649945 10.1093/hmg/ddi057

[bib96] Kwanhian W, Lenze D, Alles J, Motsch N, Barth S, Doll C et al. MicroRNA-142 is mutated in about 20% of diffuse large B-cell lymphoma. Cancer Med 2012; 1: 141–155. 23342264 10.1002/cam4.29 PMC3544448

[bib97] Verma A, Jiang Y, Du W, Fairchild L, Melnick A, Elemento O. Transcriptome sequencing reveals thousands of novel long non-coding RNAs in B cell lymphoma. Genome Med 2015; 7: 110. 26521025 10.1186/s13073-015-0230-7 PMC4628784

[bib98] Karreth FA, Reschke M, Ruocco A, Ng C, Chapuy B, Leopold V et al. The BRAF pseudogene functions as a competitive endogenous RNA and induces lymphoma *in vivo*. Cell 2015; 161: 319–332. 25843629 10.1016/j.cell.2015.02.043 PMC6922011

[bib99] Rogler LE, Kosmyna B, Moskowitz D, Bebawee R, Rahimzadeh J, Kutchko K et al. Small RNAs derived from lncRNA RNase MRP have gene-silencing activity relevant to human cartilage-hair hypoplasia. Hum Mol Genet 2014; 23: 368–382. 24009312 10.1093/hmg/ddt427 PMC3869355

[bib100] Luzon-Toro B, Bleda M, Navarro E, Garcia-Alonso L, Ruiz-Ferrer M, Medina I et al. Identification of epistatic interactions through genome-wide association studies in sporadic medullary and juvenile papillary thyroid carcinomas. BMC Med Genomics 2015; 8: 83. 26690675 10.1186/s12920-015-0160-7 PMC4685628

