# Supplementary material for: Germline mutations predisposing to diffuse large B-cell lymphoma
Source: Blood Cancer J. 2017 Feb 17;7(2):e532–. doi: 10.1038/bcj.2017.15 (PMC5386333; doi:10.1038/bcj.2017.15)
Supplement: Supplementary Information [file bcj201715x1.docx]

Supplemental file: Table s1, Phenotypic assignment of genes information, References

**Table s1. Genes with somatic mutations in DLBCL**

| **ABI3BP** | PRKDC | DUSP | SETD2 | GPR15 | DUPD1 | XIRP2 | PCDHB2 | DPF2 |
| --- | --- | --- | --- | --- | --- | --- | --- | --- |
| **TP53** | XRCC5 | **DUSP2** | SETD5 | **GPR37** | DYNC1H1 | COBL | PCDHB3 | **TBL1XR1** |
| USP7 | XRCC6 | DUSP9 | NSD1 | GPR112 | TSC22D1 | SPTBN1 | PCDHB5 | **IFNGR1** |
| RUNDC1 | NIPBL | SDCCAG1(NEMF) | **CREBBP** | GPR133 | PDE6A | SPTBN2 | **PCDHB6** | **IRF4** |
| TP63 | EXO1 | CUL4B | **HDAC7** | NPY2R | PDE1C | MYO5C | PCDHB10 | **IRF8** |
| **UNC5D** | MLH1 | CCNF | TRRAP | RGS1 | CNP^a^ | MYRIP | **PCDHB11** | WDR65 |
| TAF1 | MLH3 | MARCH7 | MN1 | LPHN2 | ARHGEF1 | FLNC | PCDHB15 | WDR66 |
| TAF1L | MSH2 | FBX031 | CRTC3 | GOPC | ARHGEF2 | CORO2A | PCDHGA2 | ANKRD17 |
| TAF4B | MSH6 | FBXW7 | EPC2 | **GNA12** | ERBB2IP | CORO7 | CELSR3 | ANKRD44 |
| ATM | POLA1 | HACE1 | EP300 | **GNA13** | **KRTAP5-5** | **ACTB** | CDH3 | ANKRD50 |
| SYNE1 | POLE | PDZRN3 | NFX1 | FRMPD1 | **SLC38A8** | ACTN1 | **CDH9** | **LYN** |
| PPP2R5A | POLG | PDZRN4 | **KLHL6** | AKAP8 | SLC16A7 | DIAPH1 | CDH10 | HCK |
| **RB1** | POLQ^a^ | TRIM37 | **KLHL14** | AKAP9 | SLC4A8 | DIAPH3 | CDH19 | BTK |
| PDCD4 | LIG3 | HISTH1C | **EZH2** | DCC | SLC5A1 | ZWILCH | PLEKHA7 | PIK3CD |
| CDK2A | **EEF1A1** | HISTH1E | WDR88 | **S1PR2** | SLC5A12 | **DNAH5** | ITGA2 | PIK3R1 |
| CDK2B | **EIF4A2** | HISTH2AL | WIF1 | **P2RY8** | **SLC25A48** | DNAH6 | ITGA8 | MTMR3 |
| CDK11A | **SRRM2** | **HISTH2BC** | APC | **P2RX5** | SLC9A5 | **DNAH8** | ITGB3 | AFAP1 |
| **CDKN2A** | MED12L | HISTH2BK | BMPR1A | GABRA1 | RHOA | DNAH10 | ODZ3 | MTMR8 |
| **CCND3** | MED13L | HISTH2BO | TGFBR3 | GABRG1 | AMIGO3 | SPEG | TLN2 | MTOR |
| NEIL1 | BTAF1 | **HIST1H1C** | CRELD2 | RELN | RHOH | KIF1 | KIAA2022 | TSC2 |
| NTHL1 | AFF2 | **HIST1H1D** | GREM2 | NLGN2 | CDC42EP1 | KIF21B | CTNNA3 | SYK |
| RAD17 | ATIC | **HIST1H1E** | KDM6A | NRXN2 | FGD3 | KLC3 | SMEK1 | MEGF10 |
| RAD51B | HNRNPR | **HIST1H2AC** | KDM2B | **NRXN3** | DOCK2 | INSC | ROR2 | **TMEM30A** |
| RAD51AP1 | ALKBH8 | **HIST1H2AG** | **OR6K3** | **CNTNAP5** | DOCK4 | MYOM2 | GJB4 | ATP10A |
| RBM15B | **ZFP36L1** | **HIST1H2BC** | OR8H3 | CHRM5 | LRP1B | **SRPX** | GJA8 | HMGB1 |
| STRADA | APOBEC2 | **HIST1H3B** | **OR10A2** | GRIA2 | LRP1 | NEB | **DCDC5** | **TNFAIP3** |
| APBB1 | DDX2B | HIST1H41 | OR51D1 | **CXCR4** | LRP2 | **TMSL3** | **SOCS1** | **DTX1** |
| APP | **DDX3X** | H1FOO | OFD1 | PTEN | LRP3 | **TMSB4X** | **STAT3** | NOTCH1 |
| GIYD1 | DDX10 | BAZ1A | RPGRIP1L | **SLITRK3** | LRP10 | FAT1 | STAT6 | NOTCH2 |
| BLM | DPYD | FRYL | SEMA3A | **SLITRK6** | OSBPL10 | **FAT2** | NFIB | JAG1 |
| BRCA2 | GCFC1 | **KMT2D(MLL2)** | IQUB | DLGAP1 | PNLIPRP1 | FAT3 | **MYD88** | ONECUT1 |
| FANCD2 | PABPC1 | MLL3 | TTC18 | IQGAP1 | BSCL2 | **FAT4** | BCR | MIB1 |
| FANCL | PABPC5 | DIP2B | RGS12 | RAB3GAP1 | APOA5 | **MPDZ** | IGSF5^a^ | ADAM2 |
| CHEK2 | CNOT6 | BRD4 | **PKD1** | **SGK1** | NCEH1 | DCHS1 | CD79A | ADAM10 |
| PARP1 | MDN1 | ARID1A | PTPRF | BRAF | DENND3 | DSC2 | **CD79B** | NRK |
| DDB1 | CDC123 | SMARCA1 | PTPRM | RAPGEF1 | RASGEF1A | DSC3 | TSPAN7 | ZP1 |
| UVRAG | DHX15 | ATRX | PTPRT | RAPGEF2 | GBP6 | DSP | **FAS** | TRAF2 |
| INTS6 | ETF1 | **MEF2B** | PPP1R9A | KRAS | DCX | DSCAML1 | HIPK3 | TRAF3 |
| MDC1 | ZNF830 | MEF2C | NPHP1 | RIMS1 | USH2A | DSG2 | TNF | TRAF5 |
| DCLRE1C | MORC2 | CIC | UNC13C | MFHAS1 | EPHA3 | **DSG4** | **CARD11** | IPTKB |
| **POGZ** | **UBE2A** | IDH1 | VPS13A | NF1 | EPHA5 | **PCDH7** | **NFKBIA** | TNFRSF11A |
| **PCLO** | WAC | TET2 | COL4A2 | ACSS3 | EPHA7 | PCDHB1 | C9 | **TNFRSF14** |
| **SYN2** | TTN | WT1 | COL4A6 | AHR | LIFR | SH3PXD2B | MBL2 | TNFSF14 |
| PRX | ABCC9 | **POSTN** | COL5A2 | ZNF521 | MPL | SHMT2 | CAPN5 | TNFSF9 |
| BRSK1 | ABCC12 | LRAP | COL22A1 | PHOX2A | NGFR | UQCRC1 | SIGLEC10 | **SARM1** |
| **Table s1. continued** | | | | | | | | |
|  |  |  |  |  |  |  |  |  |
| ATP2C2 | ABCA3 | ADH7 | **IER2** | **PASD1** | **PDGFC** | WHSC1 | **IGLL5** | SCYL1 |
| ATP2A3 | ABCA4 | ALDH1L2 | **FNDC1** | MORF4L2 | NELL2 | PADI1 | **BCL2** | TRIP11 |
| ATP8B4 | ABCA7 | XDH | LEPREL1 | HNF1B | EGF1 | PAPPA2 | BCL2L10 | LYST |
| RFC1 | ABCB1 | AGXT2L1 | FREM2 | HSF2 | **LRRN3** | PBX1 | BCL7A | ACE |
| FIGN | STOML2 | ALAS1 | LAMA1 | BTBD3 | ERBB3 | PCCB | **BCL6** | CD36 |
| GIMAP5 | CALR | AMAC1 | KRT6A | ZBTB1 | GRB2 | PDIA2 | **BCL10** | **CD58** |
| CPNE7 | RYR1 | BCAT2 | TCHH | **ETV6** | PXK | PPWD1 | **PIM1** | **CD70** |
| SCNN1A | RYR2 | SAMD9 | HMCN1 | ZFP36L1 | LRIG3 | PNPT1 | MYC | CD74 |
| CNGA4 | CAMTA1 | **SAMD9L** | FLG | ZFHX3 | LIN7C | PPARGC1A | **PRDM1** | **ODZ2** |
| ATP1A2 | FKBP9 | CYP24A1 | CNTN6 | ZNF311 | FGFR1 | **PRKCB** | PRDM15 | ROBO1 |
| AP1G2 | CPS1 | CYP2C18^a^ | ANTXR1 | ZNF394 | KLB | PRKCQ | PRDM16 | **ROBO2** |
| KCNA3 | LRRIQ3 | GPD2 | EXTL2 | ZNF91 | KDR | PROS1 | SOX6 | **UNC5C** |
| KCNAB3 | LRRC49 | H6PD | EXTL3 | ZNF366 | SEMA3D | PRSS7 | PAX5 | **WDFY3** |
| KCNA5 | CSMD1 | NDUFS1 | **DSEL** | ZNF354A | SEMA5A | PTPN14 | **ETS1** | HLA-DMB |
| KCNJ6 | CSMD3 | MUC16 | B4GALNT2 | ZNF474 | PLA2G4B | **PTPN23** | KLF2 | **CD83** |
| KCNJ12 | SPECC1 | HK3 | ST8SIA3 | ZNF608 | MYL7 | RSL1D1 | KLF4 | **B2M** |
| KCNT2 | ADCK1 | PGM2L1 | SULF2 | ZNF700 | DCBLD1 | SAPS3 | **EBF1** | TPRKB |
| WNK4 | SERPINA1 | FMO2 | TLL2 | ZIC4 | SUPT16HP | SERINC2 | **FOXO1** | UHRF1BP1L |
| EYA4 | SERPINA6 | DMXL1 | CXorf48 | TSHZ2 | EXOC6B | CCDC46 | **IKZF3** | **MPEG1** |
| TMC1 | A2ML1 | ALG13 | C12orf35 | **BTG1** | **SYPL1** | CCDC97 | MAP3K7 | NOC3L |
| ANK2 | CST7 | DOLK | MAGEC3 | **BTG2** | TMEM161A | CCDC132 | **HLA-A** | UNC13B |
| ANK3 | ADAMTSL3 | DUOXA2 | RGAG1 | **POU2F2** | TIMM50 | MYO1G | HLA-B |  |
| MYH7 | ADAMTS15 | ERN2 | ACSM3 | TDRD6 | TMEM16E | CAPN7 | **CIITA** |  |
| MYH7B | THBS4 | FAM62C | ACSS2 | KIT | TMEM63A | NLRP7 | **NLRP5** |  |
| **PIEZO2** | ENAM | GCN1L1 | SP8 | PDGFRA | WDR67 | CERC1 | LILRA2 |  |

^a^ germline variants denoted as polymorphisms

In red the 24 genes of which a (presumed) causative germline mutation has been detected in DLBCL; in blue the 211 genes of which germline alterations exist not known to predispose to DLBCL. 118 genes significantly mutated in DLBCL in bold.

Of all 626 genes somatically mutated in DLBCL 235, or over one third, may carry germline mutations. For the 118 significantly mutated in this disease 42 ( 5 plus 37), a slightly lower proportion.

**Phenotypic category assignment germline mutations of somatically mutated genes in DLBCL**

Germline mutations responsible for a phenotype other than DLBCL may not be identical to those observed somatically in DLBCL thus far. Somatic mutations detected in DLBCL were not systematically compared with mutations known to occur in the germline. An account for the category assignment of genes is provided below.

Malignancy: *UNC5C*^1^, *TRIM37*^2, 3^, *ZNF311*^4^, *FBXW7*^5^, *TSC2*^6^, *RB1*^7^,^8^ *FANCD2*^9^, *FANCL*^9^, *EXO1*^10^, *MSH2*^11^, *POLE*^12^, *IGSF5*^13^, *HNF1B*^14^, *APC*^15^, *WT1*^5^, *WIF1*^16^, *CSMD3*^17^, *NF1*^18^, *FAT4*^19^, *BMPR1A*^20^, *PTEN*^21^, *DCC*^22^, *SETD2*^23^, *PDGFRA*^24^, *POLQ*^25^, *MLL3*^26^, *NTHL1*^27^, *SOCS1*^28^, *B-RAF*^29^, *K-RAS*^30^, *CARD11*^31,32^, *ETV6*^33^, *PAX5*^34^, *A2ML1*^35^, *EZH2* ^36,37^, *ATP2A3*^38^, *CDKN2A^39^, SAMD9L^40^,SRRM2^41^*

The role of a germline variant of *CSMD3* in colorectal cancer susceptibility awaits further clarification.17 SNPs in *DCC* may confer an increased risk in cancer. True mutations cause congenital mirror movements.^42^ *A2ML1* (alpha2macroglobulin-like1) mutations were recently shown to be associated with a disorder clinically related to Noonan syndrome (NS). No hemato-oncological disease in NS patients with germline mutations in *A2ML1* have been reported yet. Inclusion of this mutation in this category is therefore premature and based solely on extrapolation of data from other Rasopathies. SAMDL9 was recently shown to be mutated in ataxia-pancytopenia syndrome. This disorder manifests itself by cerebellar ataxia, variable blood cytopenias and a propensity to develop bone marrow failure and myelogeneous leukemia.^40^ Originally reported as a cancer susceptibility disease , it illustrates the difficulty in assigning genes with known germline mutations to a single phenotypic category.

Intellectual disability: *AFF2*^43^, *KMT2D*^44^, *KCNJ6*^45^, *ANK3*^46^, *ATRX*^47^ ,*SETD5*^48^, *ATIC*^49^, *DCHS1*^50^, *DYNC1H1*^51^, *ALG13*^52^, *LRP2*^53^, *PCCB*^54^, *FBXO31*^55^, *TSPAN7*^56^, *NIPBL*^57^, *MED13L*^58^, *CUL4B*^47^, *NSD1*^59^, *RAB3GAP1*^60^, *GPD2*^61,62^, *EXOC6B*^63^, *UBE2A*^47^, *CAMTA1*^64^, *SOX6*^65^, *KIAA2022*^66^, *ACTB*^67^, *HACE1*^68^, *CREBBP*^69^, *WAC*^70^, *USP7* ^71^,*TAF1*^72^, *DDX3X*^73^, *KDM6A*^44^, *MEF2C*^74^, *PCLO^75^, PTPN23^76^*

Visual impairment: *ANTXR1*^77^, *SLC38A8*^78^, *RIMS1*^79^, *LEPREL1*^80^, *PHOX2A*^81^, *CAPN5*^82^, *MPDZ*^83^, *ABCA4*^84^, *LAMA1*^85^, *PDE6A*^86^, *GJA8*^87^, *HMCN1*^88^, *ODZ3*^89^, *ABCA3*^90^, *SRPX^91^*

Cardiomyopathy: *MIB1*^92^, *TTN*^93^, *ABCC9*^94^, *MYH7*^95^, *MYH7B*^96^, *SPEG*^97^, *DSC2*^98^, *CTNNA3*^99^, *PRDM16*^100^, *DSP*^101^, *DSG2*^102^, *XIRP2*^103^

Immunodeficiency: *BTK*^104^, *CXCR4*^105^, *CERC1*^106, 107^, *BCL10*^108^, *DOCK2*^109^, *RHOH*^110^, *CD79A*^111^, *CD79B*^112^, *IRF8*^113^, *NFKBIA^114^*, *CIITA^115^*

Renal defects: *ACE*^116^, *ROBO2*^117^, *PKD1*^118^, *RPGRIP1L*^119^, *NPHP1*^120^, *XDH*^121^, *CYP24A1*^122^, *ITGA8*^123^, *FREM2*^123^

*CYP24A1* mutations primarily cause idiopathic infantile hypercalcemia by affecting vitamin D metabolism with nefrocalcinosis as a secondary phenomenon. XDH mutations cause xanthinuria with possible secondary renal failure due to kidney stones.

Hearing loss: *SLITRK6*^124^, *PNPT1*^125^, *TMC1*^126^, *DIAPH1*^127^, *DIAPH3*^128^, *EYA4*^129^, *COL4A6*^130^, *USH2A*^131^

Seizures: *ATP1A2*^132^, *STRADA*^133^, *VPS13A*^134^, *RELN*^135^, *DOLK*^136^, *DCX*^137^, *DPYD*^138^ , *GABRA1*^139^

Skin disorder: *GJB4*^140^, *FLG*^141^, *COL5A2*^142^, *KRT6A*^143^ , *SAMD9*^144^, *ADAM10*^145^

Skeletal anomaly: *LIFR*^146^, *ROR2*^147^,^148^, *SH3PXD2B*^149^,*TMEM16E*(*GDD1*)^150^, *NOTCH2*^151^

Cardiac arrhythmia: *ZFHX3*^152^, *KCNA5*^153^, *AKAP9*^154^, *ANK2*^155^, *RYR2*^156^

Infertility: *HSF2*^157^, *TAF4B*^158^, *H6PD*^159^, *ZP1*^160^, *NLRP5^161^*

Thrombocytopenia: *ACTN1*^162^, *ITGB3*^163^, *MPL*^164^, *LYST*^165^,

Myopathy: *RYR1*^166^, *MEGF10*^167^, *FLNC*^168^, *NEB*^169^

Neuropathy: *PRX*^170^, *BSCL2*^171^, *POLG*^172^, *MORC2*^173^

Oral clefting: *OFD1*^174^, *WDR65*^175^, *MN1*^176^, *PIEZO2*(*FAM38B*)^177^

Autism: *NRXN2*^178^, *NRXN3^179^, POGZ^180^, SYN2^181^*

Ataxia: *SYNE1*^182^, *SPTBN2*^183^, *NDUFS1*^184^

Hypotrichosis: *DSC3*^185^, *CDH3*^186^, *DSG4^187^*

Pseudohypoaldosteronism: *WNK4*^188^, *SCNN1A*^189^

Hypogonadotropic hypogonadism: *FGFR1*^190^, *SEMA3A*^191^

Teeth anomalies: *ENAM*^192^, *GREM2*^193^

Dementia: *APP*^194^, *ABCA7*^195^

Schizophrenia: *TRRAP*^196^

Athelia: *PTPRF*^197^

Catatonia-depression syndrome: *CNP* polymorphism^198^

Glucose-galactose malabsorption: *SLC5A1*^199^

Hypertriglyceridemia: *APOA5*^200^

Primary ciliary dyskinesia: *DNAH5*^201^

Hyperammonemia: *CPS1*^202^

α1-antitrypsin deficiency: *SERPINA1*^203^

Corticosteroid binding globulin deficiency: *SERPINA6*^204^

Altered drug metabolism: *CYP2C18*^205^polymorphism, complete linkage with *CYP2C19* determines its effect.

Hypothyroidism: *DUOXA2*^206^

Congenital hemiplegia: *COL4A2*^207^

Total anomalous venous connection: *SEMA3D*^208^

Hereditary thrombophilia: *PROS1*^209^

Complement factor C9 deficiency: *C9*^210^

Alagille syndrome: *JAG1*^211^

Moyamoya disease: *RNF213*^212^

Hypervalinemia, hyperleucine-isoleucinemia: *BCAT2*^213^

Bicuspid aortic valve: *NOTCH1*^214^

Note added in proof: Stritt et al recently extended the phenotypic repertoire of DIAPH1 mutations by reporting a gain-of –function variant in DIAPH1 causing dominant macrothombocytopenia and hearing loss^215^

**References**

1. Coissieux MM, Tomsic J, Castets M, Hampel H, Tuupanen S, Andrieu N*, et al.* Variants in the netrin-1 receptor UNC5C prevent apoptosis and increase risk of familial colorectal cancer. *Gastroenterology* 2011; **141:** 2039-2046.

2. Hamalainen RH, Avela K, Lambert JA, Kallijarvi J, Eyaid W, Gronau J*, et al.* Novel mutations in the TRIM37 gene in Mulibrey Nanism. *Human mutation* 2004; **23:** 522.

3. Karlberg N, Karlberg S, Karikoski R, Mikkola S, Lipsanen-Nyman M, Jalanko H. High frequency of tumours in Mulibrey nanism. *The Journal of pathology* 2009; **218:** 163-171.

4. Renieri A, Mencarelli MA, Cetta F, Baldassarri M, Mari F, Furini S*, et al.* Oligogenic germline mutations identified in early non-smokers lung adenocarcinoma patients. *Lung cancer* 2014; **85:** 168-174.

5. Williams RD, Al-Saadi R, Chagtai T, Popov S, Messahel B, Sebire N*, et al.* Subtype-specific FBXW7 mutation and MYCN copy number gain in Wilms' tumor. *Clinical cancer research : an official journal of the American Association for Cancer Research* 2010; **16:** 2036-2045.

6. Kotulska K, Borkowska J, Mandera M, Roszkowski M, Jurkiewicz E, Grajkowska W*, et al.* Congenital subependymal giant cell astrocytomas in patients with tuberous sclerosis complex. *Child's nervous system : ChNS : official journal of the International Society for Pediatric Neurosurgery* 2014; **30:** 2037-2042.

7. Barbosa RH, Vargas FR, Aguiar FC, Ferman S, Lucena E, Bonvicino CR*, et al.* Hereditary retinoblastoma transmitted by maternal germline mosaicism. *Pediatric blood & cancer* 2008; **51:** 598-602.

8. Marees T, Moll AC, Imhof SM, de Boer MR, Ringens PJ, van Leeuwen FE. Risk of second malignancies in survivors of retinoblastoma: more than 40 years of follow-up. *Journal of the National Cancer Institute* 2008; **100:** 1771-1779.

9. Akbari MR, Malekzadeh R, Lepage P, Roquis D, Sadjadi AR, Aghcheli K*, et al.* Mutations in Fanconi anemia genes and the risk of esophageal cancer. *Human genetics* 2011; **129:** 573-582.

10. Wu Y, Berends MJ, Post JG, Mensink RG, Verlind E, Van Der Sluis T*, et al.* Germline mutations of EXO1 gene in patients with hereditary nonpolyposis colorectal cancer (HNPCC) and atypical HNPCC forms. *Gastroenterology* 2001; **120:** 1580-1587.

11. Sehgal R, Sheahan K, O'Connell PR, Hanly AM, Martin ST, Winter DC. Lynch syndrome: an updated review. *Genes* 2014; **5:** 497-507.

12. Palles C, Cazier JB, Howarth KM, Domingo E, Jones AM, Broderick P*, et al.* Germline mutations affecting the proofreading domains of POLE and POLD1 predispose to colorectal adenomas and carcinomas. *Nature genetics* 2013; **45:** 136-144.

13. Wang J, Carvajal-Carmona LG, Chu JH, Zauber AG, Collaborators APCT, Kubo M*, et al.* Germline variants and advanced colorectal adenomas: adenoma prevention with celecoxib trial genome-wide association study. *Clinical cancer research : an official journal of the American Association for Cancer Research* 2013; **19:** 6430-6437.

14. Rebouissou S, Vasiliu V, Thomas C, Bellanne-Chantelot C, Bui H, Chretien Y*, et al.* Germline hepatocyte nuclear factor 1alpha and 1beta mutations in renal cell carcinomas. *Human molecular genetics* 2005; **14:** 603-614.

15. Yamaguchi K, Komura M, Yamaguchi R, Imoto S, Shimizu E, Kasuya S*, et al.* Detection of APC mosaicism by next-generation sequencing in an FAP patient. *Journal of human genetics* 2015; **60:** 227-231.

16. Wei C, Peng B, Han Y, Chen WV, Rother J, Tomlinson GE*, et al.* Mutations of HNRNPA0 and WIF1 predispose members of a large family to multiple cancers. *Familial cancer* 2015; **14:** 297-306.

17. Gylfe AE, Sirkia J, Ahlsten M, Jarvinen H, Mecklin JP, Karhu A*, et al.* Somatic mutations and germline sequence variants in patients with familial colorectal cancer. *International journal of cancer Journal international du cancer* 2010; **127:** 2974-2980.

18. Alkindy A, Chuzhanova N, Kini U, Cooper DN, Upadhyaya M. Genotype-phenotype associations in neurofibromatosis type 1 (NF1): an increased risk of tumor complications in patients with NF1 splice-site mutations? *Human genomics* 2012; **6:** 12.

19. Furukawa T, Sakamoto H, Takeuchi S, Ameri M, Kuboki Y, Yamamoto T*, et al.* Whole exome sequencing reveals recurrent mutations in BRCA2 and FAT genes in acinar cell carcinomas of the pancreas. *Scientific reports* 2015; **5:** 8829.

20. Howe JR, Bair JL, Sayed MG, Anderson ME, Mitros FA, Petersen GM*, et al.* Germline mutations of the gene encoding bone morphogenetic protein receptor 1A in juvenile polyposis. *Nature genetics* 2001; **28:** 184-187.

21. Tan MH, Mester JL, Ngeow J, Rybicki LA, Orloff MS, Eng C. Lifetime cancer risks in individuals with germline PTEN mutations. *Clinical cancer research : an official journal of the American Association for Cancer Research* 2012; **18:** 400-407.

22. Rai R, Sharma KL, Tiwari S, Misra S, Kumar A, Mittal B. DCC (deleted in colorectal carcinoma) gene variants confer increased susceptibility to gallbladder cancer (Ref. No.: Gene-D-12-01446). *Gene* 2013; **518:** 303-309.

23. Yoshikawa Y, Sato A, Tsujimura T, Otsuki T, Fukuoka K, Hasegawa S*, et al.* Biallelic germline and somatic mutations in malignant mesothelioma: multiple mutations in transcription regulators including mSWI/SNF genes. *International journal of cancer Journal international du cancer* 2015; **136:** 560-571.

24. Ricci R, Martini M, Cenci T, Carbone A, Lanza P, Biondi A*, et al.* PDGFRA-mutant syndrome. *Mod Pathol* 2015; **28:** 954-964.

25. Brandalize AP, Schuler-Faccini L, Hoffmann JS, Caleffi M, Cazaux C, Ashton-Prolla P. A DNA repair variant in POLQ (c.-1060A > G) is associated to hereditary breast cancer patients: a case-control study. *BMC Cancer* 2014; **14:** 850.

26. Sasaki MM, Skol AD, Bao R, Rhodes LV, Chambers R, Vokes EE*, et al.* Integrated genomic analysis suggests MLL3 is a novel candidate susceptibility gene for familial nasopharyngeal carcinoma. *Cancer epidemiology, biomarkers & prevention : a publication of the American Association for Cancer Research, cosponsored by the American Society of Preventive Oncology* 2015; **24:** 1222-1228.

27. Weren RD, Ligtenberg MJ, Kets CM, de Voer RM, Verwiel ET, Spruijt L*, et al.* A germline homozygous mutation in the base-excision repair gene NTHL1 causes adenomatous polyposis and colorectal cancer. *Nature genetics* 2015; **47:** 668-671.

28. Mottok A, Renne C, Willenbrock K, Hansmann ML, Brauninger A. Somatic hypermutation of SOCS1 in lymphocyte-predominant Hodgkin lymphoma is accompanied by high JAK2 expression and activation of STAT6. *Blood* 2007; **110:** 3387-3390.

29. Makita Y, Narumi Y, Yoshida M, Niihori T, Kure S, Fujieda K*, et al.* Leukemia in Cardio-facio-cutaneous (CFC) syndrome: a patient with a germline mutation in BRAF proto-oncogene. *Journal of pediatric hematology/oncology* 2007; **29:** 287-290.

30. Schubbert S, Zenker M, Rowe SL, Boll S, Klein C, Bollag G*, et al.* Germline KRAS mutations cause Noonan syndrome. *Nature genetics* 2006; **38:** 331-336.

31. Snow AL, Xiao W, Stinson JR, Lu W, Chaigne-Delalande B, Zheng L*, et al.* Congenital B cell lymphocytosis explained by novel germline CARD11 mutations. *The Journal of experimental medicine* 2012; **209:** 2247-2261.

32. Brohl AS, Stinson JR, Su HC, Badgett T, Jennings CD, Sukumar G*, et al.* Germline CARD11 Mutation in a Patient with Severe Congenital B Cell Lymphocytosis. *Journal of clinical immunology* 2015; **35:** 32-46.

33. Zhang MY, Churpek JE, Keel SB, Walsh T, Lee MK, Loeb KR*, et al.* Germline ETV6 mutations in familial thrombocytopenia and hematologic malignancy. *Nature genetics* 2015; **47:** 180-185.

34. Shah S, Schrader KA, Waanders E, Timms AE, Vijai J, Miething C*, et al.* A recurrent germline PAX5 mutation confers susceptibility to pre-B cell acute lymphoblastic leukemia. *Nature genetics* 2013; **45:** 1226-1231.

35. Vissers LE, Bonetti M, Paardekooper Overman J, Nillesen WM, Frints SG, de Ligt J*, et al.* Heterozygous germline mutations in A2ML1 are associated with a disorder clinically related to Noonan syndrome. *European journal of human genetics : EJHG* 2015; **23:** 317-324.

36. Tatton-Brown K, Hanks S, Ruark E, Zachariou A, Duarte Sdel V, Ramsay E*, et al.* Germline mutations in the oncogene EZH2 cause Weaver syndrome and increased human height. *Oncotarget* 2011; **2:** 1127-1133.

37. Usemann J, Ernst T, Schafer V, Lehmberg K, Seeger K. EZH2 mutation in an adolescent with Weaver syndrome developing acute myeloid leukemia and secondary hemophagocytic lymphohistiocytosis. *American journal of medical genetics Part A* 2016; **170:** 1274-1277.

38. Korosec B, Glavac D, Volavsek M, Ravnik-Glavac M. ATP2A3 gene is involved in cancer susceptibility. *Cancer Genet Cytogenet* 2009; **188:** 88-94.

39. Monzon J, Liu L, Brill H, Goldstein AM, Tucker MA, From L*, et al.* CDKN2A mutations in multiple primary melanomas. *The New England journal of medicine* 1998; **338:** 879-887.

40. Chen DH, Below JE, Shimamura A, Keel SB, Matsushita M, Wolff J*, et al.* Ataxia-Pancytopenia Syndrome Is Caused by Missense Mutations in SAMD9L. *American journal of human genetics* 2016; **98:** 1146-1158.

41. Tomsic J, He H, Akagi K, Liyanarachchi S, Pan Q, Bertani B*, et al.* A germline mutation in SRRM2, a splicing factor gene, is implicated in papillary thyroid carcinoma predisposition. *Scientific reports* 2015; **5:** 10566.

42. Srour M, Riviere JB, Pham JM, Dube MP, Girard S, Morin S*, et al.* Mutations in DCC cause congenital mirror movements. *Science* 2010; **328:** 592.

43. Stettner GM, Shoukier M, Hoger C, Brockmann K, Auber B. Familial intellectual disability and autistic behavior caused by a small FMR2 gene deletion. *American journal of medical genetics Part A* 2011; **155A:** 2003-2007.

44. Micale L, Augello B, Maffeo C, Selicorni A, Zucchetti F, Fusco C*, et al.* Molecular analysis, pathogenic mechanisms, and readthrough therapy on a large cohort of Kabuki syndrome patients. *Human mutation* 2014; **35:** 841-850.

45. Masotti A, Uva P, Davis-Keppen L, Basel-Vanagaite L, Cohen L, Pisaneschi E*, et al.* Keppen-Lubinsky syndrome is caused by mutations in the inwardly rectifying K+ channel encoded by KCNJ6. *American journal of human genetics* 2015; **96:** 295-300.

46. Iqbal Z, Vandeweyer G, van der Voet M, Waryah AM, Zahoor MY, Besseling JA*, et al.* Homozygous and heterozygous disruptions of ANK3: at the crossroads of neurodevelopmental and psychiatric disorders. *Human molecular genetics* 2013; **22:** 1960-1970.

47. Tzschach A, Grasshoff U, Beck-Woedl S, Dufke C, Bauer C, Kehrer M*, et al.* Next-generation sequencing in X-linked intellectual disability. *European journal of human genetics : EJHG* 2015; **23:** 1513-1518.

48. Kuechler A, Zink AM, Wieland T, Ludecke HJ, Cremer K, Salviati L*, et al.* Loss-of-function variants of SETD5 cause intellectual disability and the core phenotype of microdeletion 3p25.3 syndrome. *European journal of human genetics : EJHG* 2015; **23:** 753-760.

49. Marie S, Heron B, Bitoun P, Timmerman T, Van Den Berghe G, Vincent MF. AICA-ribosiduria: a novel, neurologically devastating inborn error of purine biosynthesis caused by mutation of ATIC. *American journal of human genetics* 2004; **74:** 1276-1281.

50. Cappello S, Gray MJ, Badouel C, Lange S, Einsiedler M, Srour M*, et al.* Mutations in genes encoding the cadherin receptor-ligand pair DCHS1 and FAT4 disrupt cerebral cortical development. *Nature genetics* 2013; **45:** 1300-1308.

51. Scoto M, Rossor AM, Harms MB, Cirak S, Calissano M, Robb S*, et al.* Novel mutations expand the clinical spectrum of DYNC1H1-associated spinal muscular atrophy. *Neurology* 2015; **84:** 668-679.

52. Bissar-Tadmouri N, Donahue WL, Al-Gazali L, Nelson SF, Bayrak-Toydemir P, Kantarci S. X chromosome exome sequencing reveals a novel ALG13 mutation in a nonsyndromic intellectual disability family with multiple affected male siblings. *American journal of medical genetics Part A* 2014; **164A:** 164-169.

53. Kantarci S, Al-Gazali L, Hill RS, Donnai D, Black GC, Bieth E*, et al.* Mutations in LRP2, which encodes the multiligand receptor megalin, cause Donnai-Barrow and facio-oculo-acoustico-renal syndromes. *Nature genetics* 2007; **39:** 957-959.

54. Pena L, Franks J, Chapman KA, Gropman A, Ah Mew N, Chakrapani A*, et al.* Natural history of propionic acidemia. *Molecular genetics and metabolism* 2012; **105:** 5-9.

55. Mir A, Sritharan K, Mittal K, Vasli N, Araujo C, Jamil T*, et al.* Truncation of the E3 ubiquitin ligase component FBXO31 causes non-syndromic autosomal recessive intellectual disability in a Pakistani family. *Human genetics* 2014; **133:** 975-984.

56. Zemni R, Bienvenu T, Vinet MC, Sefiani A, Carrie A, Billuart P*, et al.* A new gene involved in X-linked mental retardation identified by analysis of an X;2 balanced translocation. *Nature genetics* 2000; **24:** 167-170.

57. Mei L, Liang D, Huang Y, Pan Q, Wu L. Two novel NIPBL gene mutations in Chinese patients with Cornelia de Lange syndrome. *Gene* 2015; **555:** 476-480.

58. Cafiero C, Marangi G, Orteschi D, Ali M, Asaro A, Ponzi E*, et al.* Novel de novo heterozygous loss-of-function variants in MED13L and further delineation of the MED13L haploinsufficiency syndrome. *European journal of human genetics : EJHG* 2015; **23:** 1499-1504.

59. Fickie MR, Lapunzina P, Gentile JK, Tolkoff-Rubin N, Kroshinsky D, Galan E*, et al.* Adults with Sotos syndrome: review of 21 adults with molecularly confirmed NSD1 alterations, including a detailed case report of the oldest person. *American journal of medical genetics Part A* 2011; **155A:** 2105-2111.

60. Picker-Minh S, Busche A, Hartmann B, Spors B, Klopocki E, Hubner C*, et al.* Large homozygous RAB3GAP1 gene microdeletion causes Warburg micro syndrome 1. *Orphanet journal of rare diseases* 2014; **9:** 113.

61. Daoud H, Gruchy N, Constans JM, Moussaoui E, Saumureau S, Bayou N*, et al.* Haploinsufficiency of the GPD2 gene in a patient with nonsyndromic mental retardation. *Human genetics* 2009; **124:** 649-658.

62. Barge-Schaapveld DQ, Ofman R, Knegt AC, Alders M, Hohne W, Kemp S*, et al.* Intellectual disability and hemizygous GPD2 mutation. *American journal of medical genetics Part A* 2013; **161A:** 1044-1050.

63. Evers C, Maas B, Koch KA, Jauch A, Janssen JW, Sutter C*, et al.* Mosaic deletion of EXOC6B: further evidence for an important role of the exocyst complex in the pathogenesis of intellectual disability. *American journal of medical genetics Part A* 2014; **164A:** 3088-3094.

64. Thevenon J, Lopez E, Keren B, Heron D, Mignot C, Altuzarra C*, et al.* Intragenic CAMTA1 rearrangements cause non-progressive congenital ataxia with or without intellectual disability. *Journal of medical genetics* 2012; **49:** 400-408.

65. Scott O, Pugh J, Kiddoo D, Sonnenberg LK, Bamforth S, Goez HR. Global developmental delay, progressive relapsing-remitting parkinsonism, and spinal syrinx in a child with SOX6 mutation. *Journal of child neurology* 2014; **29:** NP164-167.

66. Van Maldergem L, Hou Q, Kalscheuer VM, Rio M, Doco-Fenzy M, Medeira A*, et al.* Loss of function of KIAA2022 causes mild to severe intellectual disability with an autism spectrum disorder and impairs neurite outgrowth. *Human molecular genetics* 2013; **22:** 3306-3314.

67. Riviere JB, van Bon BW, Hoischen A, Kholmanskikh SS, O'Roak BJ, Gilissen C*, et al.* De novo mutations in the actin genes ACTB and ACTG1 cause Baraitser-Winter syndrome. *Nature genetics* 2012; **44:** 440-444, S441-442.

68. Hollstein R, Parry DA, Nalbach L, Logan CV, Strom TM, Hartill VL*, et al.* HACE1 deficiency causes an autosomal recessive neurodevelopmental syndrome. *Journal of medical genetics* 2015; **52:** 797-803.

69. Petrij F, Giles RH, Dauwerse HG, Saris JJ, Hennekam RC, Masuno M*, et al.* Rubinstein-Taybi syndrome caused by mutations in the transcriptional co-activator CBP. *Nature* 1995; **376:** 348-351.

70. DeSanto C, D'Aco K, Araujo GC, Shannon N, Study DDD, Vernon H*, et al.* WAC loss-of-function mutations cause a recognisable syndrome characterised by dysmorphic features, developmental delay and hypotonia and recapitulate 10p11.23 microdeletion syndrome. *Journal of medical genetics* 2015; **52:** 754-761.

71. Hao YH, Fountain MD, Jr., Fon Tacer K, Xia F, Bi W, Kang SH*, et al.* USP7 Acts as a Molecular Rheostat to Promote WASH-Dependent Endosomal Protein Recycling and Is Mutated in a Human Neurodevelopmental Disorder. *Molecular cell* 2015; **59:** 956-969.

72. O'Rawe JA, Wu Y, Dorfel MJ, Rope AF, Au PY, Parboosingh JS*, et al.* TAF1 Variants Are Associated with Dysmorphic Features, Intellectual Disability, and Neurological Manifestations. *American journal of human genetics* 2015; **97:** 922-932.

73. Snijders Blok L, Madsen E, Juusola J, Gilissen C, Baralle D, Reijnders MR*, et al.* Mutations in DDX3X Are a Common Cause of Unexplained Intellectual Disability with Gender-Specific Effects on Wnt Signaling. *American journal of human genetics* 2015; **97:** 343-352.

74. Tanteles GA, Alexandrou A, Evangelidou P, Gavatha M, Anastasiadou V, Sismani C. Partial MEF2C deletion in a Cypriot patient with severe intellectual disability and a jugular fossa malformation: review of the literature. *American journal of medical genetics Part A* 2015; **167A:** 664-669.

75. Ahmed MY, Chioza BA, Rajab A, Schmitz-Abe K, Al-Khayat A, Al-Turki S*, et al.* Loss of PCLO function underlies pontocerebellar hypoplasia type III. *Neurology* 2015; **84:** 1745-1750.

76. Alazami AM, Patel N, Shamseldin HE, Anazi S, Al-Dosari MS, Alzahrani F*, et al.* Accelerating novel candidate gene discovery in neurogenetic disorders via whole-exome sequencing of prescreened multiplex consanguineous families. *Cell Rep* 2015; **10:** 148-161.

77. Stranecky V, Hoischen A, Hartmannova H, Zaki MS, Chaudhary A, Zudaire E*, et al.* Mutations in ANTXR1 cause GAPO syndrome. *American journal of human genetics* 2013; **92:** 792-799.

78. Poulter JA, Al-Araimi M, Conte I, van Genderen MM, Sheridan E, Carr IM*, et al.* Recessive mutations in SLC38A8 cause foveal hypoplasia and optic nerve misrouting without albinism. *American journal of human genetics* 2013; **93:** 1143-1150.

79. Sisodiya SM, Thompson PJ, Need A, Harris SE, Weale ME, Wilkie SE*, et al.* Genetic enhancement of cognition in a kindred with cone-rod dystrophy due to RIMS1 mutation. *Journal of medical genetics* 2007; **44:** 373-380.

80. Mordechai S, Gradstein L, Pasanen A, Ofir R, El Amour K, Levy J*, et al.* High myopia caused by a mutation in LEPREL1, encoding prolyl 3-hydroxylase 2. *American journal of human genetics* 2011; **89:** 438-445.

81. Nakano M, Yamada K, Fain J, Sener EC, Selleck CJ, Awad AH*, et al.* Homozygous mutations in ARIX(PHOX2A) result in congenital fibrosis of the extraocular muscles type 2. *Nature genetics* 2001; **29:** 315-320.

82. Mahajan VB, Skeie JM, Bassuk AG, Fingert JH, Braun TA, Daggett HT*, et al.* Calpain-5 mutations cause autoimmune uveitis, retinal neovascularization, and photoreceptor degeneration. *PLoS genetics* 2012; **8:** e1003001.

83. Ali M, Hocking PM, McKibbin M, Finnegan S, Shires M, Poulter JA*, et al.* Mpdz null allele in an avian model of retinal degeneration and mutations in human leber congenital amaurosis and retinitis pigmentosa. *Investigative ophthalmology & visual science* 2011; **52:** 7432-7440.

84. Zhang N, Tsybovsky Y, Kolesnikov AV, Rozanowska M, Swider M, Schwartz SB*, et al.* Protein misfolding and the pathogenesis of ABCA4-associated retinal degenerations. *Human molecular genetics* 2015; **24:** 3220-3237.

85. Aldinger KA, Mosca SJ, Tetreault M, Dempsey JC, Ishak GE, Hartley T*, et al.* Mutations in LAMA1 cause cerebellar dysplasia and cysts with and without retinal dystrophy. *American journal of human genetics* 2014; **95:** 227-234.

86. Shen S, Sujirakul T, Tsang SH. Next-generation sequencing revealed a novel mutation in the gene encoding the beta subunit of rod phosphodiesterase. *Ophthalmic genetics* 2014; **35:** 142-150.

87. Mackay DS, Bennett TM, Culican SM, Shiels A. Exome sequencing identifies novel and recurrent mutations in GJA8 and CRYGD associated with inherited cataract. *Human genomics* 2014; **8:** 19.

88. Schultz DW, Klein ML, Humpert AJ, Luzier CW, Persun V, Schain M*, et al.* Analysis of the ARMD1 locus: evidence that a mutation in HEMICENTIN-1 is associated with age-related macular degeneration in a large family. *Human molecular genetics* 2003; **12:** 3315-3323.

89. Aldahmesh MA, Mohammed JY, Al-Hazzaa S, Alkuraya FS. Homozygous null mutation in ODZ3 causes microphthalmia in humans. *Genet Med* 2012; **14:** 900-904.

90. Chen P, Dai Y, Wu X, Wang Y, Sun S, Xiao J*, et al.* Mutations in the ABCA3 gene are associated with cataract-microcornea syndrome. *Investigative ophthalmology & visual science* 2014; **55:** 8031-8043.

91. Meindl A, Carvalho MR, Herrmann K, Lorenz B, Achatz H, Lorenz B*, et al.* A gene (SRPX) encoding a sushi-repeat-containing protein is deleted in patients with X-linked retinitis pigmentosa. *Human molecular genetics* 1995; **4:** 2339-2346.

92. Luxan G, Casanova JC, Martinez-Poveda B, Prados B, D'Amato G, MacGrogan D*, et al.* Mutations in the NOTCH pathway regulator MIB1 cause left ventricular noncompaction cardiomyopathy. *Nature medicine* 2013; **19:** 193-201.

93. Herman DS, Lam L, Taylor MR, Wang L, Teekakirikul P, Christodoulou D*, et al.* Truncations of titin causing dilated cardiomyopathy. *The New England journal of medicine* 2012; **366:** 619-628.

94. Bienengraeber M, Olson TM, Selivanov VA, Kathmann EC, O'Cochlain F, Gao F*, et al.* ABCC9 mutations identified in human dilated cardiomyopathy disrupt catalytic KATP channel gating. *Nature genetics* 2004; **36:** 382-387.

95. Fananapazir L, Dalakas MC, Cyran F, Cohn G, Epstein ND. Missense mutations in the beta-myosin heavy-chain gene cause central core disease in hypertrophic cardiomyopathy. *Proceedings of the National Academy of Sciences of the United States of America* 1993; **90:** 3993-3997.

96. Mora R, Merino JL, Peinado R, Olias F, Garcia-Guereta L, del Cerro MJ*, et al.* [Hypertrophic cardiomyopathy: infrequent mutation of the cardiac beta-myosin heavy-chain gene]. *Revista espanola de cardiologia* 2006; **59:** 846-849.

97. Agrawal PB, Pierson CR, Joshi M, Liu X, Ravenscroft G, Moghadaszadeh B*, et al.* SPEG interacts with myotubularin, and its deficiency causes centronuclear myopathy with dilated cardiomyopathy. *American journal of human genetics* 2014; **95:** 218-226.

98. Zhou X, Chen M, Song H, Wang B, Chen H, Wang J*, et al.* Comprehensive analysis of desmosomal gene mutations in Han Chinese patients with arrhythmogenic right ventricular cardiomyopathy. *European journal of medical genetics* 2015; **58:** 258-265.

99. van Hengel J, Calore M, Bauce B, Dazzo E, Mazzotti E, De Bortoli M*, et al.* Mutations in the area composita protein alphaT-catenin are associated with arrhythmogenic right ventricular cardiomyopathy. *European heart journal* 2013; **34:** 201-210.

100. Arndt AK, Schafer S, Drenckhahn JD, Sabeh MK, Plovie ER, Caliebe A*, et al.* Fine mapping of the 1p36 deletion syndrome identifies mutation of PRDM16 as a cause of cardiomyopathy. *American journal of human genetics* 2013; **93:** 67-77.

101. Pigors M, Schwieger-Briel A, Cosgarea R, Diaconeasa A, Bruckner-Tuderman L, Fleck T*, et al.* Desmoplakin mutations with palmoplantar keratoderma, woolly hair and cardiomyopathy. *Acta dermato-venereologica* 2015; **95:** 337-340.

102. Pereira Fernandes M, Azevedo O, Pereira V, Calvo L, Lourenco A. Arrhythmogenic right ventricular cardiomyopathy with left ventricular involvement: a novel splice site mutation in the DSG2 gene. *Cardiology* 2015; **130:** 159-161.

103. Long PA, Larsen BT, Evans JM, Olson TM. Exome Sequencing Identifies Pathogenic and Modifier Mutations in a Child With Sporadic Dilated Cardiomyopathy. *J Am Heart Assoc* 2015; **4**.

104. Winkelstein JA, Marino MC, Lederman HM, Jones SM, Sullivan K, Burks AW*, et al.* X-linked agammaglobulinemia: report on a United States registry of 201 patients. *Medicine* 2006; **85:** 193-202.

105. Al Ustwani O, Kurzrock R, Wetzler M. Genetics on a WHIM. *British journal of haematology* 2014; **164:** 15-23.

106. Navon Elkan P, Pierce SB, Segel R, Walsh T, Barash J, Padeh S*, et al.* Mutant adenosine deaminase 2 in a polyarteritis nodosa vasculopathy. *The New England journal of medicine* 2014; **370:** 921-931.

107. Zhou Q, Yang D, Ombrello AK, Zavialov AV, Toro C, Zavialov AV*, et al.* Early-onset stroke and vasculopathy associated with mutations in ADA2. *The New England journal of medicine* 2014; **370:** 911-920.

108. Torres JM, Martinez-Barricarte R, Garcia-Gomez S, Mazariegos MS, Itan Y, Boisson B*, et al.* Inherited BCL10 deficiency impairs hematopoietic and nonhematopoietic immunity. *The Journal of clinical investigation* 2014; **124:** 5239-5248.

109. Dobbs K, Dominguez Conde C, Zhang SY, Parolini S, Audry M, Chou J*, et al.* Inherited DOCK2 Deficiency in Patients with Early-Onset Invasive Infections. *The New England journal of medicine* 2015; **372:** 2409-2422.

110. Crequer A, Troeger A, Patin E, Ma CS, Picard C, Pedergnana V*, et al.* Human RHOH deficiency causes T cell defects and susceptibility to EV-HPV infections. *The Journal of clinical investigation* 2012; **122:** 3239-3247.

111. Minegishi Y, Coustan-Smith E, Rapalus L, Ersoy F, Campana D, Conley ME. Mutations in Igalpha (CD79a) result in a complete block in B-cell development. *The Journal of clinical investigation* 1999; **104:** 1115-1121.

112. Dobbs AK, Yang T, Farmer D, Kager L, Parolini O, Conley ME. Cutting edge: a hypomorphic mutation in Igbeta (CD79b) in a patient with immunodeficiency and a leaky defect in B cell development. *Journal of immunology* 2007; **179:** 2055-2059.

113. Hambleton S, Salem S, Bustamante J, Bigley V, Boisson-Dupuis S, Azevedo J*, et al.* IRF8 mutations and human dendritic-cell immunodeficiency. *The New England journal of medicine* 2011; **365:** 127-138.

114. Courtois G, Smahi A, Reichenbach J, Doffinger R, Cancrini C, Bonnet M*, et al.* A hypermorphic IkappaBalpha mutation is associated with autosomal dominant anhidrotic ectodermal dysplasia and T cell immunodeficiency. *The Journal of clinical investigation* 2003; **112:** 1108-1115.

115. Steimle V, Otten LA, Zufferey M, Mach B. Complementation cloning of an MHC class II transactivator mutated in hereditary MHC class II deficiency (or bare lymphocyte syndrome). *Cell* 1993; **75:** 135-146.

116. Danilov SM, Kalinin S, Chen Z, Vinokour EI, Nesterovitch AB, Schwartz DE*, et al.* Angiotensin I-converting enzyme Gln1069Arg mutation impairs trafficking to the cell surface resulting in selective denaturation of the C-domain. *PloS one* 2010; **5:** e10438.

117. Lu W, van Eerde AM, Fan X, Quintero-Rivera F, Kulkarni S, Ferguson H*, et al.* Disruption of ROBO2 is associated with urinary tract anomalies and confers risk of vesicoureteral reflux. *American journal of human genetics* 2007; **80:** 616-632.

118. Ali H, Hussain N, Naim M, Zayed M, Al-Mulla F, Kehinde EO*, et al.* A novel PKD1 variant demonstrates a disease-modifying role in trans with a truncating PKD1 mutation in patients with Autosomal Dominant Polycystic Kidney Disease. *BMC nephrology* 2015; **16:** 26.

119. Delous M, Baala L, Salomon R, Laclef C, Vierkotten J, Tory K*, et al.* The ciliary gene RPGRIP1L is mutated in cerebello-oculo-renal syndrome (Joubert syndrome type B) and Meckel syndrome. *Nature genetics* 2007; **39:** 875-881.

120. Hildebrandt F, Otto E, Rensing C, Nothwang HG, Vollmer M, Adolphs J*, et al.* A novel gene encoding an SH3 domain protein is mutated in nephronophthisis type 1. *Nature genetics* 1997; **17:** 149-153.

121. Gok F, Ichida K, Topaloglu R. Mutational analysis of the xanthine dehydrogenase gene in a Turkish family with autosomal recessive classical xanthinuria. *Nephrology, dialysis, transplantation : official publication of the European Dialysis and Transplant Association - European Renal Association* 2003; **18:** 2278-2283.

122. Schlingmann KP, Kaufmann M, Weber S, Irwin A, Goos C, John U*, et al.* Mutations in CYP24A1 and idiopathic infantile hypercalcemia. *The New England journal of medicine* 2011; **365:** 410-421.

123. Kohl S, Hwang DY, Dworschak GC, Hilger AC, Saisawat P, Vivante A*, et al.* Mild recessive mutations in six Fraser syndrome-related genes cause isolated congenital anomalies of the kidney and urinary tract. *Journal of the American Society of Nephrology : JASN* 2014; **25:** 1917-1922.

124. Tekin M, Chioza BA, Matsumoto Y, Diaz-Horta O, Cross HE, Duman D*, et al.* SLITRK6 mutations cause myopia and deafness in humans and mice. *The Journal of clinical investigation* 2013; **123:** 2094-2102.

125. von Ameln S, Wang G, Boulouiz R, Rutherford MA, Smith GM, Li Y*, et al.* A mutation in PNPT1, encoding mitochondrial-RNA-import protein PNPase, causes hereditary hearing loss. *American journal of human genetics* 2012; **91:** 919-927.

126. Kurima K, Peters LM, Yang Y, Riazuddin S, Ahmed ZM, Naz S*, et al.* Dominant and recessive deafness caused by mutations of a novel gene, TMC1, required for cochlear hair-cell function. *Nature genetics* 2002; **30:** 277-284.

127. Lynch ED, Lee MK, Morrow JE, Welcsh PL, Leon PE, King MC. Nonsyndromic deafness DFNA1 associated with mutation of a human homolog of the Drosophila gene diaphanous. *Science* 1997; **278:** 1315-1318.

128. Schoen CJ, Emery SB, Thorne MC, Ammana HR, Sliwerska E, Arnett J*, et al.* Increased activity of Diaphanous homolog 3 (DIAPH3)/diaphanous causes hearing defects in humans with auditory neuropathy and in Drosophila. *Proceedings of the National Academy of Sciences of the United States of America* 2010; **107:** 13396-13401.

129. Wayne S, Robertson NG, DeClau F, Chen N, Verhoeven K, Prasad S*, et al.* Mutations in the transcriptional activator EYA4 cause late-onset deafness at the DFNA10 locus. *Human molecular genetics* 2001; **10:** 195-200.

130. Rost S, Bach E, Neuner C, Nanda I, Dysek S, Bittner RE*, et al.* Novel form of X-linked nonsyndromic hearing loss with cochlear malformation caused by a mutation in the type IV collagen gene COL4A6. *European journal of human genetics : EJHG* 2014; **22:** 208-215.

131. Eudy JD, Weston MD, Yao S, Hoover DM, Rehm HL, Ma-Edmonds M*, et al.* Mutation of a gene encoding a protein with extracellular matrix motifs in Usher syndrome type IIa. *Science* 1998; **280:** 1753-1757.

132. Deprez L, Weckhuysen S, Peeters K, Deconinck T, Claeys KG, Claes LR*, et al.* Epilepsy as part of the phenotype associated with ATP1A2 mutations. *Epilepsia* 2008; **49:** 500-508.

133. Galanopoulou AS, Gorter JA, Cepeda C. Finding a better drug for epilepsy: the mTOR pathway as an antiepileptogenic target. *Epilepsia* 2012; **53:** 1119-1130.

134. Rampoldi L, Dobson-Stone C, Rubio JP, Danek A, Chalmers RM, Wood NW*, et al.* A conserved sorting-associated protein is mutant in chorea-acanthocytosis. *Nature genetics* 2001; **28:** 119-120.

135. Guerrini R, Filippi T. Neuronal migration disorders, genetics, and epileptogenesis. *Journal of child neurology* 2005; **20:** 287-299.

136. Lieu MT, Ng BG, Rush JS, Wood T, Basehore MJ, Hegde M*, et al.* Severe, fatal multisystem manifestations in a patient with dolichol kinase-congenital disorder of glycosylation. *Molecular genetics and metabolism* 2013; **110:** 484-489.

137. Lawrence KM, Mei D, Newton MR, Leventer RJ, Guerrini R, Berkovic SF. Familial Lennox-Gastaut syndrome in male siblings with a novel DCX mutation and anterior pachygyria. *Epilepsia* 2010; **51:** 1902-1905.

138. van Kuilenburg AB, Meijer J, Gokcay G, Baykal T, Rubio-Gozalbo ME, Mul AN*, et al.* Dihydropyrimidine dehydrogenase deficiency caused by a novel genomic deletion c.505_513del of DPYD. *Nucleosides, nucleotides & nucleic acids* 2010; **29:** 509-514.

139. Epi KC, Epilepsy Phenome/Genome P, Allen AS, Berkovic SF, Cossette P, Delanty N*, et al.* De novo mutations in epileptic encephalopathies. *Nature* 2013; **501:** 217-221.

140. Kokotas H, Papagiannaki K, Grigoriadou M, Petersen MB, Katsarou A. Erythrokeratodermia variabilis: report of two cases and a novel missense variant in GJB4 encoding connexin 30.3. *European journal of dermatology : EJD* 2012; **22:** 182-186.

141. Palmer CN, Irvine AD, Terron-Kwiatkowski A, Zhao Y, Liao H, Lee SP*, et al.* Common loss-of-function variants of the epidermal barrier protein filaggrin are a major predisposing factor for atopic dermatitis. *Nature genetics* 2006; **38:** 441-446.

142. Ritelli M, Dordoni C, Venturini M, Chiarelli N, Quinzani S, Traversa M*, et al.* Clinical and molecular characterization of 40 patients with classic Ehlers-Danlos syndrome: identification of 18 COL5A1 and 2 COL5A2 novel mutations. *Orphanet journal of rare diseases* 2013; **8:** 58.

143. Wilson NJ, Leachman SA, Hansen CD, McMullan AC, Milstone LM, Schwartz ME*, et al.* A large mutational study in pachyonychia congenita. *The Journal of investigative dermatology* 2011; **131:** 1018-1024.

144. Topaz O, Indelman M, Chefetz I, Geiger D, Metzker A, Altschuler Y*, et al.* A deleterious mutation in SAMD9 causes normophosphatemic familial tumoral calcinosis. *American journal of human genetics* 2006; **79:** 759-764.

145. Kono M, Sugiura K, Suganuma M, Hayashi M, Takama H, Suzuki T*, et al.* Whole-exome sequencing identifies ADAM10 mutations as a cause of reticulate acropigmentation of Kitamura, a clinical entity distinct from Dowling-Degos disease. *Human molecular genetics* 2013; **22:** 3524-3533.

146. Dagoneau N, Scheffer D, Huber C, Al-Gazali LI, Di Rocco M, Godard A*, et al.* Null leukemia inhibitory factor receptor (LIFR) mutations in Stuve-Wiedemann/Schwartz-Jampel type 2 syndrome. *American journal of human genetics* 2004; **74:** 298-305.

147. Afzal AR, Rajab A, Fenske CD, Oldridge M, Elanko N, Ternes-Pereira E*, et al.* Recessive Robinow syndrome, allelic to dominant brachydactyly type B, is caused by mutation of ROR2. *Nature genetics* 2000; **25:** 419-422.

148. Oldridge M, Fortuna AM, Maringa M, Propping P, Mansour S, Pollitt C*, et al.* Dominant mutations in ROR2, encoding an orphan receptor tyrosine kinase, cause brachydactyly type B. *Nature genetics* 2000; **24:** 275-278.

149. Iqbal Z, Cejudo-Martin P, de Brouwer A, van der Zwaag B, Ruiz-Lozano P, Scimia MC*, et al.* Disruption of the podosome adaptor protein TKS4 (SH3PXD2B) causes the skeletal dysplasia, eye, and cardiac abnormalities of Frank-Ter Haar Syndrome. *American journal of human genetics* 2010; **86:** 254-261.

150. Tsutsumi S, Kamata N, Vokes TJ, Maruoka Y, Nakakuki K, Enomoto S*, et al.* The novel gene encoding a putative transmembrane protein is mutated in gnathodiaphyseal dysplasia (GDD). *American journal of human genetics* 2004; **74:** 1255-1261.

151. Simpson MA, Irving MD, Asilmaz E, Gray MJ, Dafou D, Elmslie FV*, et al.* Mutations in NOTCH2 cause Hajdu-Cheney syndrome, a disorder of severe and progressive bone loss. *Nature genetics* 2011; **43:** 303-305.

152. Tsai CT, Hsieh CS, Chang SN, Chuang EY, Juang JM, Lin LY*, et al.* Next-generation sequencing of nine atrial fibrillation candidate genes identified novel de novo mutations in patients with extreme trait of atrial fibrillation. *Journal of medical genetics* 2015; **52:** 28-36.

153. Olson TM, Alekseev AE, Liu XK, Park S, Zingman LV, Bienengraeber M*, et al.* Kv1.5 channelopathy due to KCNA5 loss-of-function mutation causes human atrial fibrillation. *Human molecular genetics* 2006; **15:** 2185-2191.

154. Chopra N, Knollmann BC. Genetics of sudden cardiac death syndromes. *Current opinion in cardiology* 2011; **26:** 196-203.

155. Mohler PJ, Schott JJ, Gramolini AO, Dilly KW, Guatimosim S, duBell WH*, et al.* Ankyrin-B mutation causes type 4 long-QT cardiac arrhythmia and sudden cardiac death. *Nature* 2003; **421:** 634-639.

156. Wanguemert F, Bosch Calero C, Perez C, Campuzano O, Beltran-Alvarez P, Scornik FS*, et al.* Clinical and molecular characterization of a cardiac ryanodine receptor founder mutation causing catecholaminergic polymorphic ventricular tachycardia. *Heart rhythm : the official journal of the Heart Rhythm Society* 2015; **12:** 1636-1643.

157. Mou L, Wang Y, Li H, Huang Y, Jiang T, Huang W*, et al.* A dominant-negative mutation of HSF2 associated with idiopathic azoospermia. *Human genetics* 2013; **132:** 159-165.

158. Ayhan O, Balkan M, Guven A, Hazan R, Atar M, Tok A*, et al.* Truncating mutations in TAF4B and ZMYND15 causing recessive azoospermia. *Journal of medical genetics* 2014; **51:** 239-244.

159. Lavery GG, Walker EA, Tiganescu A, Ride JP, Shackleton CH, Tomlinson JW*, et al.* Steroid biomarkers and genetic studies reveal inactivating mutations in hexose-6-phosphate dehydrogenase in patients with cortisone reductase deficiency. *The Journal of clinical endocrinology and metabolism* 2008; **93:** 3827-3832.

160. Huang HL, Lv C, Zhao YC, Li W, He XM, Li P*, et al.* Mutant ZP1 in familial infertility. *The New England journal of medicine* 2014; **370:** 1220-1226.

161. Docherty LE, Rezwan FI, Poole RL, Turner CL, Kivuva E, Maher ER*, et al.* Mutations in NLRP5 are associated with reproductive wastage and multilocus imprinting disorders in humans. *Nat Commun* 2015; **6:** 8086.

162. Kunishima S, Okuno Y, Yoshida K, Shiraishi Y, Sanada M, Muramatsu H*, et al.* ACTN1 mutations cause congenital macrothrombocytopenia. *American journal of human genetics* 2013; **92:** 431-438.

163. Nurden AT, Pillois X, Fiore M, Alessi MC, Bonduel M, Dreyfus M*, et al.* Expanding the Mutation Spectrum Affecting alphaIIbbeta3 Integrin in Glanzmann Thrombasthenia: Screening of the ITGA2B and ITGB3 Genes in a Large International Cohort. *Human mutation* 2015; **36:** 548-561.

164. Ihara K, Ishii E, Eguchi M, Takada H, Suminoe A, Good RA*, et al.* Identification of mutations in the c-mpl gene in congenital amegakaryocytic thrombocytopenia. *Proceedings of the National Academy of Sciences of the United States of America* 1999; **96:** 3132-3136.

165. Nagle DL, Karim MA, Woolf EA, Holmgren L, Bork P, Misumi DJ*, et al.* Identification and mutation analysis of the complete gene for Chediak-Higashi syndrome. *Nature genetics* 1996; **14:** 307-311.

166. Bharucha-Goebel DX, Santi M, Medne L, Zukosky K, Dastgir J, Shieh PB*, et al.* Severe congenital RYR1-associated myopathy: the expanding clinicopathologic and genetic spectrum. *Neurology* 2013; **80:** 1584-1589.

167. Logan CV, Lucke B, Pottinger C, Abdelhamed ZA, Parry DA, Szymanska K*, et al.* Mutations in MEGF10, a regulator of satellite cell myogenesis, cause early onset myopathy, areflexia, respiratory distress and dysphagia (EMARDD). *Nature genetics* 2011; **43:** 1189-1192.

168. Guergueltcheva V, Peeters K, Baets J, Ceuterick-de Groote C, Martin JJ, Suls A*, et al.* Distal myopathy with upper limb predominance caused by filamin C haploinsufficiency. *Neurology* 2011; **77:** 2105-2114.

169. Wallgren-Pettersson C, Lehtokari VL, Kalimo H, Paetau A, Nuutinen E, Hackman P*, et al.* Distal myopathy caused by homozygous missense mutations in the nebulin gene. *Brain : a journal of neurology* 2007; **130:** 1465-1476.

170. Boerkoel CF, Takashima H, Stankiewicz P, Garcia CA, Leber SM, Rhee-Morris L*, et al.* Periaxin mutations cause recessive Dejerine-Sottas neuropathy. *American journal of human genetics* 2001; **68:** 325-333.

171. Windpassinger C, Auer-Grumbach M, Irobi J, Patel H, Petek E, Horl G*, et al.* Heterozygous missense mutations in BSCL2 are associated with distal hereditary motor neuropathy and Silver syndrome. *Nature genetics* 2004; **36:** 271-276.

172. Tang S, Wang J, Lee NC, Milone M, Halberg MC, Schmitt ES*, et al.* Mitochondrial DNA polymerase gamma mutations: an ever expanding molecular and clinical spectrum. *Journal of medical genetics* 2011; **48:** 669-681.

173. Albulym OM, Kennerson ML, Harms MB, Drew AP, Siddell AH, Auer-Grumbach M*, et al.* MORC2 mutations cause axonal Charcot-Marie-Tooth disease with pyramidal signs. *Ann Neurol* 2016; **79:** 419-427.

174. Bisschoff IJ, Zeschnigk C, Horn D, Wellek B, Riess A, Wessels M*, et al.* Novel mutations including deletions of the entire OFD1 gene in 30 families with type 1 orofaciodigital syndrome: a study of the extensive clinical variability. *Human mutation* 2013; **34:** 237-247.

175. Rorick NK, Kinoshita A, Weirather JL, Peyrard-Janvid M, de Lima RL, Dunnwald M*, et al.* Genomic strategy identifies a missense mutation in WD-repeat domain 65 (WDR65) in an individual with Van der Woude syndrome. *American journal of medical genetics Part A* 2011; **155A:** 1314-1321.

176. Beck M, Peterson JF, McConnell J, McGuire M, Asato M, Losee JE*, et al.* Craniofacial abnormalities and developmental delay in two families with overlapping 22q12.1 microdeletions involving the MN1 gene. *American journal of medical genetics Part A* 2015; **167A:** 1047-1053.

177. McMillin MJ, Beck AE, Chong JX, Shively KM, Buckingham KJ, Gildersleeve HI*, et al.* Mutations in PIEZO2 cause Gordon syndrome, Marden-Walker syndrome, and distal arthrogryposis type 5. *American journal of human genetics* 2014; **94:** 734-744.

178. Gauthier J, Siddiqui TJ, Huashan P, Yokomaku D, Hamdan FF, Champagne N*, et al.* Truncating mutations in NRXN2 and NRXN1 in autism spectrum disorders and schizophrenia. *Human genetics* 2011; **130:** 563-573.

179. Vaags AK, Lionel AC, Sato D, Goodenberger M, Stein QP, Curran S*, et al.* Rare deletions at the neurexin 3 locus in autism spectrum disorder. *American journal of human genetics* 2012; **90:** 133-141.

180. Fukai R, Hiraki Y, Yofune H, Tsurusaki Y, Nakashima M, Saitsu H*, et al.* A case of autism spectrum disorder arising from a de novo missense mutation in POGZ. *Journal of human genetics* 2015; **60:** 277-279.

181. Corradi A, Fadda M, Piton A, Patry L, Marte A, Rossi P*, et al.* SYN2 is an autism predisposing gene: loss-of-function mutations alter synaptic vesicle cycling and axon outgrowth. *Human molecular genetics* 2014; **23:** 90-103.

182. Gros-Louis F, Dupre N, Dion P, Fox MA, Laurent S, Verreault S*, et al.* Mutations in SYNE1 lead to a newly discovered form of autosomal recessive cerebellar ataxia. *Nature genetics* 2007; **39:** 80-85.

183. Ikeda Y, Dick KA, Weatherspoon MR, Gincel D, Armbrust KR, Dalton JC*, et al.* Spectrin mutations cause spinocerebellar ataxia type 5. *Nature genetics* 2006; **38:** 184-190.

184. Benit P, Chretien D, Kadhom N, de Lonlay-Debeney P, Cormier-Daire V, Cabral A*, et al.* Large-scale deletion and point mutations of the nuclear NDUFV1 and NDUFS1 genes in mitochondrial complex I deficiency. *American journal of human genetics* 2001; **68:** 1344-1352.

185. Ayub M, Basit S, Jelani M, Ur Rehman F, Iqbal M, Yasinzai M*, et al.* A homozygous nonsense mutation in the human desmocollin-3 (DSC3) gene underlies hereditary hypotrichosis and recurrent skin vesicles. *American journal of human genetics* 2009; **85:** 515-520.

186. Sprecher E, Bergman R, Richard G, Lurie R, Shalev S, Petronius D*, et al.* Hypotrichosis with juvenile macular dystrophy is caused by a mutation in CDH3, encoding P-cadherin. *Nature genetics* 2001; **29:** 134-136.

187. Kljuic A, Bazzi H, Sundberg JP, Martinez-Mir A, O'Shaughnessy R, Mahoney MG*, et al.* Desmoglein 4 in hair follicle differentiation and epidermal adhesion: evidence from inherited hypotrichosis and acquired pemphigus vulgaris. *Cell* 2003; **113:** 249-260.

188. Wilson FH, Disse-Nicodeme S, Choate KA, Ishikawa K, Nelson-Williams C, Desitter I*, et al.* Human hypertension caused by mutations in WNK kinases. *Science* 2001; **293:** 1107-1112.

189. Wang J, Yu T, Yin L, Li J, Yu L, Shen Y*, et al.* Novel mutations in the SCNN1A gene causing Pseudohypoaldosteronism type 1. *PloS one* 2013; **8:** e65676.

190. Dode C, Levilliers J, Dupont JM, De Paepe A, Le Du N, Soussi-Yanicostas N*, et al.* Loss-of-function mutations in FGFR1 cause autosomal dominant Kallmann syndrome. *Nature genetics* 2003; **33:** 463-465.

191. Young J, Metay C, Bouligand J, Tou B, Francou B, Maione L*, et al.* SEMA3A deletion in a family with Kallmann syndrome validates the role of semaphorin 3A in human puberty and olfactory system development. *Human reproduction* 2012; **27:** 1460-1465.

192. Mardh CK, Backman B, Holmgren G, Hu JC, Simmer JP, Forsman-Semb K. A nonsense mutation in the enamelin gene causes local hypoplastic autosomal dominant amelogenesis imperfecta (AIH2). *Human molecular genetics* 2002; **11:** 1069-1074.

193. Kantaputra PN, Kaewgahya M, Hatsadaloi A, Vogel P, Kawasaki K, Ohazama A*, et al.* GREMLIN 2 Mutations and Dental Anomalies. *Journal of dental research* 2015; **94:** 1646-1652.

194. Loy CT, Schofield PR, Turner AM, Kwok JB. Genetics of dementia. *Lancet* 2014; **383:** 828-840.

195. Steinberg S, Stefansson H, Jonsson T, Johannsdottir H, Ingason A, Helgason H*, et al.* Loss-of-function variants in ABCA7 confer risk of Alzheimer's disease. *Nature genetics* 2015; **47:** 445-447.

196. Xu B, Ionita-Laza I, Roos JL, Boone B, Woodrick S, Sun Y*, et al.* De novo gene mutations highlight patterns of genetic and neural complexity in schizophrenia. *Nature genetics* 2012; **44:** 1365-1369.

197. Borck G, de Vries L, Wu HJ, Smirin-Yosef P, Nurnberg G, Lagovsky I*, et al.* Homozygous truncating PTPRF mutation causes athelia. *Human genetics* 2014; **133:** 1041-1047.

198. Hagemeyer N, Goebbels S, Papiol S, Kastner A, Hofer S, Begemann M*, et al.* A myelin gene causative of a catatonia-depression syndrome upon aging. *EMBO Mol Med* 2012; **4:** 528-539.

199. Vallaeys L, Van Biervliet S, De Bruyn G, Loeys B, Moring AS, Van Deynse E*, et al.* Congenital glucose-galactose malabsorption: a novel deletion within the SLC5A1 gene. *European journal of pediatrics* 2013; **172:** 409-411.

200. Priore Oliva C, Pisciotta L, Li Volti G, Sambataro MP, Cantafora A, Bellocchio A*, et al.* Inherited apolipoprotein A-V deficiency in severe hypertriglyceridemia. *Arterioscler Thromb Vasc Biol* 2005; **25:** 411-417.

201. Olbrich H, Haffner K, Kispert A, Volkel A, Volz A, Sasmaz G*, et al.* Mutations in DNAH5 cause primary ciliary dyskinesia and randomization of left-right asymmetry. *Nature genetics* 2002; **30:** 143-144.

202. Klaus V, Vermeulen T, Minassian B, Israelian N, Engel K, Lund AM*, et al.* Highly variable clinical phenotype of carbamylphosphate synthetase 1 deficiency in one family: an effect of allelic variation in gene expression? *Clinical genetics* 2009; **76:** 263-269.

203. Ferrarotti I, Carroll TP, Ottaviani S, Fra AM, O'Brien G, Molloy K*, et al.* Identification and characterisation of eight novel SERPINA1 Null mutations. *Orphanet journal of rare diseases* 2014; **9:** 172.

204. Simard M, Hill LA, Lewis JG, Hammond GL. Naturally occurring mutations of human corticosteroid-binding globulin. *The Journal of clinical endocrinology and metabolism* 2015; **100:** E129-139.

205. Mizugaki M, Hiratsuka M, Agatsuma Y, Matsubara Y, Fujii K, Kure S*, et al.* Rapid detection of CYP2C18 genotypes by real-time fluorescence polymerase chain reaction. *J Pharm Pharmacol* 2000; **52:** 199-205.

206. Zamproni I, Grasberger H, Cortinovis F, Vigone MC, Chiumello G, Mora S*, et al.* Biallelic inactivation of the dual oxidase maturation factor 2 (DUOXA2) gene as a novel cause of congenital hypothyroidism. *The Journal of clinical endocrinology and metabolism* 2008; **93:** 605-610.

207. Yoneda Y, Haginoya K, Arai H, Yamaoka S, Tsurusaki Y, Doi H*, et al.* De novo and inherited mutations in COL4A2, encoding the type IV collagen alpha2 chain cause porencephaly. *American journal of human genetics* 2012; **90:** 86-90.

208. Degenhardt K, Singh MK, Aghajanian H, Massera D, Wang Q, Li J*, et al.* Semaphorin 3d signaling defects are associated with anomalous pulmonary venous connections. *Nature medicine* 2013; **19:** 760-765.

209. Reitsma PH, Ploos van Amstel HK, Bertina RM. Three novel mutations in five unrelated subjects with hereditary protein S deficiency type I. *The Journal of clinical investigation* 1994; **93:** 486-492.

210. Horiuchi T, Nishizaka H, Kojima T, Sawabe T, Niho Y, Schneider PM*, et al.* A non-sense mutation at Arg95 is predominant in complement 9 deficiency in Japanese. *Journal of immunology* 1998; **160:** 1509-1513.

211. Oda T, Elkahloun AG, Pike BL, Okajima K, Krantz ID, Genin A*, et al.* Mutations in the human Jagged1 gene are responsible for Alagille syndrome. *Nature genetics* 1997; **16:** 235-242.

212. Kamada F, Aoki Y, Narisawa A, Abe Y, Komatsuzaki S, Kikuchi A*, et al.* A genome-wide association study identifies RNF213 as the first Moyamoya disease gene. *Journal of human genetics* 2011; **56:** 34-40.

213. Wang XL, Li CJ, Xing Y, Yang YH, Jia JP. Hypervalinemia and hyperleucine-isoleucinemia caused by mutations in the branched-chain-amino-acid aminotransferase gene. *J Inherit Metab Dis* 2015; **38:** 855-861.

214. Garg V, Muth AN, Ransom JF, Schluterman MK, Barnes R, King IN*, et al.* Mutations in NOTCH1 cause aortic valve disease. *Nature* 2005; **437:** 270-274.

215. Stritt S, Nurden P, Turro E, Greene D, Jansen SB, Westbury SK*, et al.* A gain-of-function variant in DIAPH1 causes dominant macrothrombocytopenia and hearing loss. *Blood* 2016; **127:** 2903-2914.
